# Interventions to Optimize Medication Management in Older Adults: An Umbrella Review of Systematic Reviews

**DOI:** 10.1111/jgs.70561

**Published:** 2026-06-24

**Authors:** Saibal Das, Sanskriti Shukla, Rajesh Aadityan, Kristina Skender, Deepasree Sukumaran, Jerin Jose Cherian, Mihir Bhatta, Ashish Pathak, Samiran Panda, Santanu Kumar Tripathi, Cecilia Stålsby Lundborg

**Affiliations:** ^1^ Department of Global Public Health Karolinska Institutet Stockholm Sweden; ^2^ Indian Council of Medical Research National Institute for Research in Bacterial Infections Kolkata India; ^3^ Academy of Scientific and Innovative Research (An Institution of National Importance Established by an Act of Parliament) Ghaziabad India; ^4^ Translational Health Science and Technology Institute Faridabad India; ^5^ All India Institute of Medical Sciences Raebareli India; ^6^ Department of Pharmacology All India Institute of Medical Sciences Kalyani India; ^7^ Indian Council of Medical Research New Delhi India; ^8^ Department of Pediatrics RD Gardi Medical College Ujjain India; ^9^ Jagannath Gupta Institute of Medical Sciences and Hospital Kolkata India

**Keywords:** deprescribing, interventions, medication management, medication optimization, medication review, older adults, polypharmacy

## Abstract

**Objective:**

Medication management in older adults with multimorbidity and polypharmacy is inherently challenging. This umbrella review consolidates evidence on interventions aimed at mitigating these challenges to promote safe and effective medication use.

**Methods:**

This was an umbrella review. A systematic search was conducted on PubMed, Embase, Scopus, Web of Science, and Cochrane CENTRAL until 2024 to find relevant systematic reviews of interventions to improve medication management among older adults aged ≥ 60 years (PROSPERO ID: CRD42024607956). Eligible systematic reviews focused on pharmacist‐led reviews, deprescribing protocols, educational programs, clinical decision support systems (CDSS), and community‐based initiatives. Data were extracted, and methodological quality was evaluated using the AMSTAR‐2 tool. Descriptive statistics were applied, and the credibility of the evidence was determined. Outcomes included medication optimization, clinical, healthcare utilization and cost, and acceptability of the intervention among physicians and patients.

**Results:**

Seventy‐one systematic reviews with > 1.5 million cumulative participants were included. According to the AMSTAR‐2 assessment, the included systematic reviews demonstrated variable methodological quality, with many rated as moderate to high risk of bias. Pharmacist‐led reviews and deprescribing interventions both indicated significant improvement in medication appropriateness. Multidisciplinary approaches and CDSS could improve adherence and prescribing practices. Improvements in clinical outcomes, such as quality of life, cognitive function, and mortality, were inconsistent. Economic evaluations showed mixed results. Implementation challenges were identified, including scalability and resource allocation. Evidence was convincing in reducing the number of medications, inappropriate prescribing, and falls.

**Conclusions:**

Pharmacist‐led and multidisciplinary medication reviews showed the most consistent benefits, improving medication appropriateness and reducing falls and unplanned healthcare use. Interventions like education, policy and guideline measures, and community‐based strategies demonstrated mixed or modest effects, often limited to adherence or prescribing quality. Overall evidence strength was limited by methodological heterogeneity. Future high‐quality, context‐specific research with standardized outcomes is needed in this regard.

## Summary

- Key Points
  ○ Pharmacist‐led and multidisciplinary medication reviews consistently improved medication appropriateness and reduced potentially inappropriate prescribing in older adults.
  ○ Deprescribing and clinical decision support systems‐based interventions showed benefits in reducing medication burden and falls, although effects on mortality and quality of life were inconsistent.
  ○ Evidence quality varied considerably across reviews, highlighting the need for standardized, high‐quality, context‐specific intervention research.
- Why Does This Paper Matter?
  ○ Medication‐related harm remains a major challenge in older adults with multimorbidity and polypharmacy. This umbrella review identifies the most effective intervention strategies and highlights critical evidence gaps to guide future clinical practice, policy, and research.

## 1 Introduction

Medication management in older adults presents a multifaceted challenge. Older adults, due to age‐related physiological changes and multimorbidity, are vulnerable to the adverse effects of inappropriate medication use [1, 2]. Polypharmacy, often defined as the concurrent use of five or more medications, is common in this population [1, 2]. While sometimes necessary, excessive or inappropriate polypharmacy elevates risks of adverse drug reactions, drug–drug interactions, prescribing cascades, medication nonadherence, and poor clinical outcomes [3, 4]. Several systematic reviews have examined various medication optimization strategies in older adults, such as pharmacist‐led interventions, deprescribing, education, and decision support tools. However, these reviews often focus on individual intervention types or settings and vary widely in methods, outcomes, quality, and strength of evidence [4, 5, 6]. For example, pharmacist‐led interventions, among other deprescribing strategies, have been shown to reduce inappropriate prescribing and enhance adherence, contributing to improved patient outcomes [7, 8]. Despite their efficacy, these interventions face barriers, such as limited healthcare provider time, lack of awareness, and resistance from patients or caregivers [9].

Recent advancements in digital health have introduced innovative solutions to address these barriers [10]. For instance, artificial intelligence‐driven clinical decision support systems (CDSS) are increasingly utilized to identify potentially inappropriate medications (PIMs) and recommend safer alternatives [10]. However, the implementation of such technologies includes disparities in digital literacy among older populations and concerns about data privacy [11]. This umbrella review consolidated evidence on interventions aimed at medication optimization to inform strategies that promote safer and more effective medication use. We adapted the definition by the World Health Organization (WHO) and frameworks, where medication management refers to a set of patient‐centered services, including medication review, reconciliation, adherence support, deprescribing, and monitoring, designed to optimize therapeutic outcomes and minimize medication‐related harm in older adults [12].

## 2 Methods

### 2.1 Search Methods

We conducted a comprehensive literature search across MEDLINE via PubMed, Embase via Elsevier, Cochrane CENTRAL via the Cochrane Library, Scopus, and Web of Science from inception to December 31, 2024. The terms were developed around three core concepts (Table S1):

- Older adults (e.g., aged, older persons, or persons aged ≥ 60 years).
- Medication optimization (e.g., polypharmacy, deprescribing, medication review, or clinical decision support).
- Systematic reviews/meta‐analyses.

Boolean operators, truncation, and proximity operators were used to refine sensitivity and specificity. No language or date restrictions were applied. References of included reviews were manually screened to identify additional eligible studies.

### 2.2 Selection of Systematic Reviews (Eligibility Criteria)

The Rayyan software was used for screening reviews. Systematic reviews, with or without meta‐analyses, were considered eligible if they evaluated any intervention(s), such as medication review, deprescribing, use of tools or devices, implementing guidelines, education, counseling, CDSS, etc., for optimizing medication management in older adults (aged ≥ 60 years). Systematic review was operationally defined as a research synthesis that employed explicit, pre‐specified methods to comprehensively identify, critically appraise, and synthesize all relevant studies addressing a specific research question. The comparators were standard care or any other intervention(s). Articles that centered around specific disease conditions (e.g., hypertension, diabetes, cancer, or dementia) or specific types of medicines (e.g., antibiotics, proton pump inhibitors, opioids, psychotropic medicines, pre‐operative medicines, anticholinergic medicines) and qualitative systematic reviews were excluded. Where systematic reviews had been updated, only the most recent versions were included. The outcomes were grouped into four types:

- Medication optimization: number of medications, number of PIMs, number of potential prescribing omissions (PPOs), medication appropriateness, adverse drug reactions or medication‐related problems, and medication adherence
- Clinical: all‐cause mortality, QoL, physical functions, falls, cognitive function, duration of hospitalization, hospital visits, admissions, and re‐admissions.
- Healthcare utilization and cost
- Acceptability of the intervention among physicians and patients.

### 2.3 Evaluation of the Quality of the Included Systematic Reviews

The methodological quality of each included systematic review was independently assessed by two reviewers (SD and SS) using the AMSTAR‐2 (A Measurement Tool to Assess Systematic Reviews) checklist [13]. Discrepancies were resolved through discussion. We extracted any GRADE (Grading of Recommendations, Assessment, Development and Evaluations) [14] assessments as reported in the included systematic reviews, which reflect the certainty of the evidence for key outcomes (e.g., high, moderate, low, or very low confidence). We did not perform independent GRADE assessments, and we did not appraise the primary studies included within each systematic review, as this was already the responsibility of the original review authors. To further evaluate the credibility of the synthesized evidence, we adopted a framework based on previously published umbrella reviews that categorize evidence strength into five levels: convincing, highly suggestive, suggestive, weak, or not significant, depending on effect size, heterogeneity, and consistency across reviews [15].

### 2.4 Data Extraction and Synthesis

Titles retrieved from search results were reviewed by two independent reviewers (SS and RA). The inclusion criteria were refined before proceeding with abstract and full‐text reviews. Disagreements were resolved by consensus with a senior reviewer (SD). Google Translate was used to translate systematic reviews published in languages other than English (Spanish and Japanese). Two reviewers (SS and RA) independently extracted data from each included systematic review using a pre‐designed, standardized abstraction sheet. Disagreements were resolved through discussion with senior reviewers (SD and DS). From each included systematic review, we extracted key characteristics, including the first author, year of publication, and number of primary studies included. We recorded details of the study populations, types of interventions evaluated, comparators used, and the outcome domains assessed. Information on the healthcare setting, key findings, and conclusions was collected.

Given the substantial heterogeneity across the included systematic reviews in terms of intervention types, outcome domains, population characteristics, and healthcare settings, a quantitative synthesis or re‐analysis of effect sizes was not feasible. Therefore, we conducted a structured narrative synthesis organized by outcome domain and intervention type. Where available, we reported effect estimates from individual reviews to illustrate the direction and magnitude of effects, but did not combine them analytically. The WHO Health Intervention Classification Framework categories were used to classify the interventions as follows: [16, 17]

- Education and training: activities aimed at improving knowledge and skills.
- Health services: direct healthcare services provided to individuals.
- Policy and guidelines: implementation of policies or guidelines to improve health outcomes.
- Community‐based: activities that engage community members in health promotion and disease prevention.

The most favorable reported effect estimates for each pairing of intervention category and outcome domain were enlisted considering the highest methodological quality of the corresponding reviews (AMSTAR‐2 criteria).

### 2.5 Handling of Overlapping Reviews or Synthesis Methods

Given the heterogeneity of included systematic reviews, a narrative synthesis approach was used. We did not perform de‐duplication or reweighting of the overlapping primary studies. Instead, we included all eligible systematic reviews, even when they addressed similar research questions. Where multiple reviews included similar sets of primary studies, we noted the potential for overlap but retained them to preserve the diversity in intervention types, settings, populations, and outcomes. A pairwise percent for the overlap metric representing the proportion of smaller reviews that are contained within the larger one was constructed. The corrected covered area (CCA) [18] was calculated using the following formula (Python version 3.13.7):

$$\mathrm{CCA}=\frac{N_{r}-N_{S}}{\left(N_{C}\times N_{S}\right)-N_{S}}$$

where $N_{r}$ is the total number of primary‐study occurrences across reviews, $N_{s}$ is the number of unique primary studies, and $N_{c}$ is the number of reviews. CCA is interpreted in banded cut‐offs: ≤ 5% is minimal overlap, 6%–10% is moderate overlap, 11%–15% is high overlap, and > 15% is very high overlap [18]. An intervention‐outcome heatmap highlighting the major intervention categories and their corresponding outcomes was also constructed. The construction of the overlap heatmap required the generation of a binary citation matrix using the *pandas* library (Python version 3.13.7), where columns denote the included systematic reviews and rows represent the primary studies. CCA was then calculated for each pairwise combination of reviews to quantify the degree of overlap, and the resulting matrix was visualized as a heatmap using libraries, such as seaborn and matplotlib (Python version 3.13.7).

### 2.6 Study Protocol and Reporting

This umbrella review complies with the preferred reporting items for overviews of reviews (PRIOR) guidelines [19]. The study protocol was registered in the International Prospective Register of Systematic Reviews (PROSPERO ID: CRD42024607956).

## 3 Results

### 3.1 Study Selection and Search Flow

**FIGURE 1:**
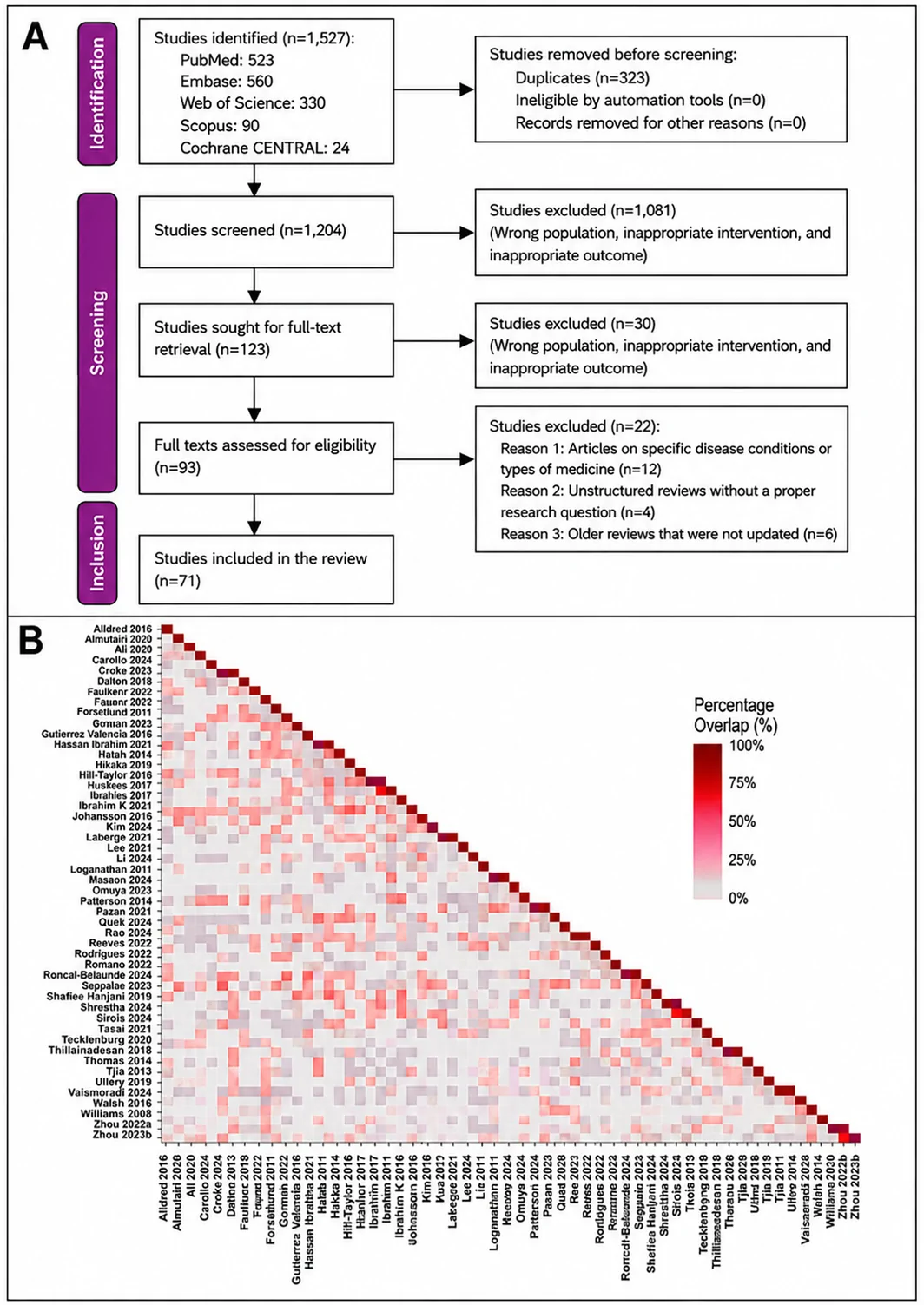
Study flowchart (preferred reporting items for overviews of reviews [PRIOR]) (A) and a pairwise percent for the overlap metric representing the proportion of smaller reviews that are contained within the larger one (B).

A total of 1527 systematic reviews were identified, and 71^s1‐s71^ were finally included (Figure 1A).

### 3.2 Overlapping Studies

Figure 1B presents the pairwise citation overlap matrix across all included systematic reviews, with overlap expressed as CCA. Overall, the CCA was 2.52%, indicating a low degree of overlap and suggesting that the evidence base was largely nonredundant. Most review pairs showed no shared citations, with 1493 of 2485 unique pairs (60.1%) having zero overlap. Only a small number of pairs demonstrated substantial overlap, including six pairs with CCA values of > 50% and five pairs with complete overlap (100%). These higher overlaps were mainly observed among reviews addressing closely related medication‐related outcomes, populations, or time periods. The citation matrix is provided in Data S1; individual cell values indicate the number of primary studies shared between each pair of systematic reviews.

### 3.3 Characteristics of the Included Systematic Reviews

The systematic reviews included a range of original study types, including randomized controlled trials, quasi‐experimental studies, and observational studies (Table 1 and Table S2). The studies were conducted in diverse settings, such as hospitals, community‐based care, nursing homes, and outpatient clinics. The cumulative sample size was > 1.5 million. The interventions were mainly delivered across a variety of healthcare contexts: hospitals/inpatient care, primary/community care, and long‐term care facilities. The majority of interventions were led by pharmacists, general practitioners, or multidisciplinary teams. The follow‐up periods varied from 1 month to 5 years. The studies primarily included older adults with frailty or multimorbidity. Overall, 28 reviews fully met all AMSTAR‐2 criteria, demonstrating strong methodological rigor, whereas 16 reviews failed to meet any criterion, indicating consistently low quality (Table S3). Additionally, 20 reviews were rated “partial” across all items, reflecting moderate but incomplete adherence to best practices. The remaining seven reviews showed mixed patterns of “yes,” “no,” and “partial” ratings, suggesting uneven methodological robustness within individual reviews.

**TABLE 1:** Summarized characteristics of the included systematic reviews.

Aspect	Summary
Designs of included studies	A majority of the systematic reviews included randomized controlled trials and cluster randomized controlled trials. Many systematic reviews also integrated quasi‐experimental studies, interrupted time‐series, prospective/retrospective cohorts, pre‐post designs, qualitative studies, mixed‐methods, and cost‐effectiveness analyses.
Settings	Broadly across the healthcare continuum: hospitals (inpatient and emergency), primary care, nursing homes, residential aged‐care, pharmacies, outpatient clinics, long‐term care, home‐based care, geriatric units, community health centers, and palliative/end‐of‐life settings. Several reviews addressed transitional care (e.g., discharge to community).
Populations	Older adults aged ≥ 60 years, often with polypharmacy (≥ 5 medications). Conditions: frail elderly, multimorbidity, dementia, palliative/end‐of‐life patients, and residents of long‐term care facilities. The cumulative sample size was > 1.5 million. Geographic reach included North America, Europe, Asia, and low‐ and middle‐income countries.
Interventions	Education and training: educational outreach programs, training modules for healthcare professionals, CDSS implementation, STOPP‐START criteria training, pharmacist‐led education. Health services: pharmacist‐led medication reviews, deprescribing initiatives, multidisciplinary care team integration, geriatric assessments, coordinated patient care models, and medication scheduling tools Policy and guidelines: STOPP‐START criteria implementation, adoption of CDSS tools, organizational reforms in medication optimization, regulatory oversight, and incentivized prescribing practices Community‐based interventions: counseling sessions, reminder systems, monitoring tools, simplified regimens, educational outreach programs for patients, telephone follow‐ups, and collaborative care models
Comparators	Most reviews compared interventions to standard care, usual prescribing, or routine practice. Some trials compared different active strategies (e.g., pharmacist vs. physician‐led review, CDSS vs. education).
Commonly reported primary outcomes	Medication appropriateness (PIMs, PPOs, medication burden index), adherence, ADRs, hospital admissions, emergency visits, mortality, falls, QoL, frailty and cognition measures, and costs.
Follow‐up duration	Ranged from short‐term (1–3 months in hospital discharge or emergency visit contexts) to medium (6–12 months in primary/community care) and long‐term (up to 5 years in nursing home and cohort studies).

Abbreviations: ADRs, adverse drug reactions; CDSS, clinical decision support system; MAI, Medication Appropriateness Index; PIMs, potentially inappropriate medications; PPOs, potential prescribing omissions; QoL, quality of life; STOPP‐START, screening tool of older Persons' prescriptions and screening tool to alert to right treatment.

### 3.4 Intervention Settings and Characteristics

A wide range of interventions were reported. Based on synthesis and mapping, we grouped them into the following major relevant categories using the WHO Health Intervention Classification Framework, although there were some overlaps (Table 2):

- Education and training: These include educational outreach programs, structured training modules for prescribers and pharmacists, implementation‐focused training on CDSS, and competency‐building sessions on deprescribing tools such as the STOPP‐START criteria. Pharmacist‐led educational initiatives also play a key role in reinforcing safe prescribing practices and enhancing medication literacy.
- Health services: These comprise pharmacist‐led medication reviews, structured deprescribing initiatives, multidisciplinary team‐based care (involving physicians, nurses, pharmacists, and geriatric specialists), and comprehensive geriatric assessments. Additional examples are coordinated patient‐care models, follow‐up services, and the use of medication scheduling tools to streamline therapy plans and support treatment adherence
- Policy and guidelines: They include implementation of STOPP‐START criteria and adoption of CDSS‐based prescribing support, as well as institutional reforms. Regulatory oversight, quality‐assurance mechanisms, and incentivized prescribing policies further support the reduction of inappropriate medication use and enhance accountability within healthcare systems.
- Community‐based: They encompass counseling sessions, reminder systems, home‐based or remote monitoring tools, and simplifying medication regimens to improve adherence. Patient education programs, telephone follow‐ups, and collaborative care models that often engage caregivers, community pharmacists, or primary care workers support continuity of care and help sustain medication‐management behaviors.

**TABLE 2:** Summary of all interventions and outcomes based on the World Health Organization health intervention classification framework categories.

Intervention classification	Intervention type	Positive outcomes	Negative outcomes	Uncertain outcomes
Education and training	Educational outreach programs, training modules for healthcare professionals, CDSS implementation, STOPP‐START criteria training, pharmacist‐led education	Significant reduction in adverse drug reactions, enhancement in medication adherence, improved identification and resolution of PIMs, better quality of life metrics	Limited evidence on reduction in all‐cause mortality, no significant impact on hospitalization duration or healthcare utilization, uncertain evidence on cognitive function improvements	Mixed results on long‐term patient satisfaction, financial sustainability, and healthcare provider acceptability
Health services	Pharmacist‐led medication reviews, deprescribing initiatives, multidisciplinary care team integration, geriatric assessments, coordinated patient care models, and medication scheduling tools	Significant improvement in medication appropriateness, enhanced adherence rates, reduction in falls and adverse events, better patient satisfaction outcomes, and reduced hospital visits	No significant improvement in cognitive and physical functions, uncertain evidence in cost‐effectiveness, inconsistent results on quality‐of‐life metrics	Economic impact varies across regions, conflicting results on resource utilization
Policy and guidelines	STOPP‐START criteria implementation, adoption of CDSS tools, organizational reforms in medication optimization, regulatory oversight, and incentivized prescribing practices	Reduction in PIMs, fewer adverse events, enhanced prescribing practices, moderate improvements in medication costs	No significant change in hospitalization rates, duration of stay, or patient mortality, uncertain evidence on long‐term clinical effectiveness	Mixed outcomes in cost‐effectiveness analysis, variable patient compliance with guidelines
Community‐based interventions	Counseling sessions, reminder systems, monitoring tools, simplified regimens, educational outreach programs for patients, telephone follow‐ups, and collaborative care models	Improved medication adherence, enhanced patient quality of life and satisfaction, better outcomes in managing community health indicators	Limited evidence in reducing adverse drug events, uncertain benefits in cost‐effectiveness, inconsistent results in clinical outcomes	Scalability and integration challenges, mixed findings on economic outcomes and healthcare provider acceptance

Abbreviations: CDSS, clinical decision support system; PIMS, potentially inappropriate medications; STOPP‐START, screening tool of older Persons' prescriptions and screening tool to alert to right treatment.

### 3.5 Outcomes Reported

Table 3 and Table S4 integrate all the systematic reviews on interventions targeting medication outcomes. Medication review and deprescribing interventions significantly reduced the number of medications, PIMs, and PPOs, and enhanced medication appropriateness. However, their effect on adverse drug reactions or medication‐related problems, as well as medication adherence, varied, with a few reports supporting consistent beneficial effects. Likewise, the impact on clinical outcomes, such as QoL, mortality, falls, and hospitalization, was variable. There were inconsistencies in the results of functional outcomes, such as mobility and cognitive improvements. Healthcare utilization metrics, including hospital admissions and re‐admissions, were modestly reduced in some reviews. Economic evaluations were noted to be cost‐effective in some contexts, although the results were inconsistent. Half of the systematic reviews failed to show any significant cost‐effectiveness. The acceptability of the interventions was found to be good among both physicians and patients. However, there were challenges in implementing the interventions in terms of scalability and resource requirements. Effective strategies to enhance medication‐related outcomes, including multidisciplinary approaches and combined interventions (e.g., medication review, multidisciplinary case‐conferencing, healthcare professional education, and CDSS), were likely to provide better results than isolated interventions.

**TABLE 3:** Summarized findings on various outcomes.

Outcome domain	Findings
Medication optimization	Number of medications: Multiple reviews confirm reductions, particularly with pharmacist or multidisciplinary deprescribing. Some reviews showed minimal or no change (context dependency); **Convincing** PIMs: strong and consistent reductions across numerous reviews. Tools such as STOPP/START and Beers criteria were effective; **Convincing** PPOs: Some improvements noted, though evidence is less robust; **Weak** Medication appropriateness: frequently improved, especially with structured review and decision‐support interventions; **Convincing** Medication adherence: Improvements were noted with patient education, counseling, reminder systems, or pharmacist‐led follow‐up; **Convincing**
Clinical	ADRs/medication‐related problems: Several reviews found meaningful reductions, though heterogeneity across settings is high; **Highly suggestive** Mortality: generally unaffected; few modest improvements; **Weak** Falls: Most reviews showed no effect; limited exceptions reported reductions in fall‐risk medication deprescribing trials; **Convincing** QoL: inconsistent, with small gains in some; **Suggestive** Cognition and physical function: limited but emerging evidence of benefit from integrated multidisciplinary deprescribing; **Not significant to weak**
Healthcare utilization and cost	Hospital admissions/readmissions: mixed reductions reported in some reviews, but null results in others; **Highly suggestive** Duration of hospitalization: Inconsistent effects were noted; **Weak** Utilization overall: Several reviews suggest system‐level benefits, though results vary by country, healthcare setting, and intervention intensity; **Weak** Cost‐effectiveness: strong support in some reviews for cost‐effective or cost‐saving impact of structured deprescribing; **Weak**
Acceptability of the intervention among physicians and patients	Physicians: Broadly positive toward pharmacist‐led and CDSS interventions; acceptance facilitated by multidisciplinary teamwork; **Suggestive** Patients: High satisfaction and willingness to deprescribe when supported by healthcare professionals. Acceptance rates commonly > 80%, demonstrating strong feasibility; **Suggestive**

*Note:* The credibility of evidence (Table S5) against each outcome is depicted in bold.

Abbreviations: ADRs: adverse drug reactions, PIMs: potentially inappropriate medications, PPOs: potential prescribing omissions, QoL: quality of life, STOPP‐START: screening tool of older Persons' prescriptions and screening tool to alert to right treatment.

### 3.6 Summary of Intervention Effects

Table 4 summarizes the most favorable reported effect estimates for each pairing of intervention category and outcome domain. Each cell cites the systematic review that reported the most positive or beneficial effect estimate, as per the methodological quality of the included reviews (AMSTAR‐2 criteria; Table S3). Clear differences emerged in the effectiveness and credibility of evidence across intervention categories. Health‐service interventions showed the most coherent and best‐supported benefits. For example, Zhou, et al., 2023^s71^ demonstrated convincing reductions in PIMs (odds ratio [OR]: 0.51, 95% confidence interval [CI]: 0.42 to 0.62), while Kim, et al., 2024^s30^ reported convincing evidence for reduced risk of all‐cause mortality (relative risk [RR]: 0.57, 95% CI: 0.37 to 0.88) and falls (RR: 0.57, 95% CI: 0.41 to 0.81). Bülow, et al., 2023^s5^ reported a reduction in hospital visits/readmissions (RR: 0.93, 95% CI: 0.89 to 0.98). These findings confirm that pharmacist‐led and multidisciplinary medication reviews provide the most reliable improvements across medication‐related, selected clinical, and healthcare utilization outcomes. Education and training interventions showed more modest effects. Page, et al., 2016^s45^ showed a significant reduction in the number of medications (mean difference [MD]: −0.99 [−1.83 to −0.14]) and PIMs (MD: −0.49 [−0.70 to −0.28]). Policy‐ and guideline‐based approaches, including CDSS, have consistently reduced inappropriate medications (Dalton, et al., 2018^s14^), although their broader effects on mortality, QoL, and healthcare utilization remained inconsistent. Community‐based strategies, such as counseling, reminder systems, and monitoring tools, were generally feasible and acceptable to patients, yet the impact on clinical outcomes was small or uncertain.

**TABLE 4:** Most favorable reported effect estimates for each pairing of intervention category and outcome domain were enlisted considering the highest methodological quality of the corresponding reviews (AMSTAR‐2 criteria).

Intervention/outcome	Medication optimization	Clinical	Health‐care utilization and cost	Acceptability of the intervention among physicians and patients
Education and training	Page, et al., 2016^s45^: Significant reduction in the number of medications [MD: −0.99 (−1.83 to −0.14)] and PIMs [MD: −0.49 (−0.70 to −0.28)]; **Convincing**	Kua, et al., 2019^s31^: All‐cause mortality (RR: 0.90, 95% CI: 0.82 to 0.99) reduced; **Weak**	Cross, et al., 2020^s13^: Hospital visits/readmissions reduced (RR: 0.67, 95% CI: 0.50 to 0.90); **Highly suggestive**	Reeve, et al., 2022^s51^: Clinician/patient uptake reported as acceptable (no pooled metric); **Suggestive**
Health services	Zhou, et al., 2023^s71^: PIMs reduced (OR: 0.51, 95% CI: 0.42 to 0.62); number of medications reduced (MD −0.94, 95% CI: −1.51 to −0.36); **Convincing**	Kim, et al., 2024^s30^: All‐cause mortality (RR: 0.57, 95% CI: 0.37 to 0.88) reduced; **Weak**, and number of falls (RR: 0.57, 95% CI: 0.41 to 0.81) reduced; **Convincing**	Bülow, et al., 2023^s5^: Hospital visits/readmissions reduced (RR: 0.93, 95% CI: 0.89 to 0.98); **Highly suggestive**	Ibrahim, et al., 2021^s28^: Well accepted by clinicians/patients (qualitative); **Suggestive**
Policy and guidelines	Dalton, et al., 2018^s14^: CDSS reduced PIMs (OR: 0.6.95% CI: 0.38 to 0.93); **Convincing**	Bloomfield, et al., 2020^s4^: All‐cause mortality (RR: 0.74, 95% CI: 0.58 to 0.959) reduced; **Weak**	Romano, et al., 2022^s53^: Cost‐effectiveness: small but significant QoL gains; most policy‐linked optimization packages were cost‐effective; **Weak**	Mixed provider compliance with guideline use; no pooled acceptability metric; **Suggestive**
Community‐based interventions	No consistent improvement in terms of medication optimization; **Weak**	No consistent improvement in clinical outcomes across reviews; **Weak**	Economic benefits mixed; no consistent pooled effect on utilization; **Weak**	Hikaka, et al., 2019^s25^: High patient satisfaction reported (qualitative); **Suggestive**

*Note:* The credibility of evidence (Table S5) against each outcome is depicted in bold.

Abbreviations: CDSS: clinical decision support system, CI: confidence interval, OR: odds ratio, MD: mean difference, PIMs: potentially inappropriate medications, RR: relative risk.

### 3.7 Certainty and Credibility of the Evidence

The GRADE of outcomes varied, and a considerable number of studies reported low to very low GRADE of evidence for different outcomes (Table S4). Table S5 summarizes the credibility of the evidence for each outcome. Outcomes, such as reduction in medications, inappropriate prescribing, and falls, as well as improvement in adherence and appropriateness, have convincing evidence. Highly suggestive evidence supports reductions in adverse reactions and hospital visits. QoL and intervention acceptability are supported by suggestive evidence. However, mortality, cognitive function, cost‐effectiveness, hospitalization, and healthcare utilization are supported by weak evidence. Physical functions showed no significant evidence.

## 4 Discussion

Medication management in older adults requires tailored, multidisciplinary strategies [20, 21, 22, 23]. This umbrella review synthesizes evidence from 71 systematic reviews to establish which intervention strategies most effectively optimize medication management in this population. Despite significant heterogeneity across reviews, a number of consistent patterns emerged. Health‐service interventions, especially pharmacist‐led and multidisciplinary medication reviews, had the most reliable improvements in medication optimization outcomes, including reductions in polypharmacy, PIMs, and PPOs. Evidence for clinical outcomes was more mixed; some reviews indicated reduced falls and adverse drug events, whereas findings for QoL, cognitive function, and mortality varied or were of low certainty. Education and training interventions demonstrated convincing improvements in medication optimization, such as reductions in total medications and PIMs, yet produced only weak evidence for mortality benefits, though they meaningfully reduced healthcare use and were generally acceptable to clinicians and patients. Policy and guideline interventions, such as CDSS and deprescribing guidance, also convincingly reduced inappropriate prescribing and fall‐risk medications, though their effects on clinical outcomes like mortality and cost‐effectiveness were weaker and more variable; acceptability findings were suggestive but lacked pooled metrics. Community‐based interventions demonstrated the weakest overall effects, with inconsistent results for medication or clinical outcomes and mixed economic benefits, although qualitative evidence suggested high patient satisfaction.

In relation to existing literature, there is an earlier scoping review of 581 medication discontinuation trials conducted in which heterogeneous methods were identified. The results suggest an imperative call for evidence‐based methodological standards in planning and conducting studies of deprescribing so that studies will be planned and conducted uniformly with reliability [24]. Another systematic review of 36 deprescribing trials focused on the importance of clinical and implementation outcomes in scaling up deprescribing practice effectively for real‐world settings [25]. Earlier overviews of systematic reviews of interventions to address polypharmacy showed that deprescribing could lower PIMs and PPOs, and mortality in end‐of‐life care. However, there was a small effect on clinical outcomes, requiring more robust evidence in various settings. There was no consistent evidence of any influence on downstream patient‐centered outcomes, for example, healthcare utilization, frailty, hospitalization, mortality, or treatment cost [26, 27, 28, 29].

The quality of evidence on deprescribing across the literature varies, and there is a need for targeted, population‐specific interventions and further research [30]. Our group had earlier reported that the overall prevalence of polypharmacy among older adults is high [31]. It was reported in a previous study that it was not possible to determine the most effective intervention to optimize medication management among older adults [32]. Combined interventions (e.g., medication review, multidisciplinary case‐conferencing, healthcare professional education, and CDSS) are likely to provide better results than isolated interventions. At the same time, there is a dearth of implementation research data related to the effectiveness of various interventions across various settings and contexts [33]. However, we found that pharmacist‐led interventions, and notably structured medication reviews along with multidisciplinary deprescribing approaches, achieve the most consistent benefits in medication optimization outcomes, which include a reduction in PIMs, PPOs, and the overall medication burden.

Our umbrella review highlights several key gaps in the current evidence base. First, although a wide variety of interventions were previously evaluated, many reviews lacked detailed reporting of intervention components, including the intensity, frequency, duration, and content of each element. This lack of granularity makes it difficult to assess fidelity, replicability, and generalizability across settings. Second, while multicomponent interventions (e.g., pharmacist‐led reviews combined with provider education or digital support) were frequently reported and appear promising in terms of improving medication appropriateness and adherence, the evidence is heterogeneous and often drawn from reviews rated as low or critically low quality. The relative contribution of each component within multicomponent packages remains unclear, and the interventions were rarely evaluated head‐to‐head. Reports on context‐specific adaptations were missing. Third, the included reviews infrequently reported standardized outcome measures, especially in domains such as QoL, cost‐effectiveness, and acceptability, which limits cross‐study comparability.

Interventions to optimize medication management among older adults vary in their complexity, applicability, and usability, and no gold standard is identifiable. Recently, we reported that deprescribing preventive medications in frail or palliative older adults was not associated with worse outcomes [34]. Explicit, criteria‐based medication reviews appear advisable for practical reasons. Considering the underuse of polypharmacy management in older adults, individual stakeholders as well as policymakers should further strengthen their efforts to promote these activities more vigorously [35]. Future research should design intervention trials with clear and standardized reporting of intervention components, compare individual vs. multicomponent approaches, using factorial or sequential designs where feasible, and evaluate interventions in diverse real‐world settings, focusing on clinical and economic outcomes [36].

The strength of our study is the inclusion of 71 systematic reviews with > 1.5 million participants in the cumulative sample size. Various interventions were included along with diverse meaningful outcomes. This updated collective data provides a current, complete picture of the evidence, identifying consistent patterns and emerging strategies that can inform current and future practices. Another strength of this review is the use of a best‐review approach rather than simple review counts, ensuring that conclusions are based on the highest‐quality evidence for each intervention‐outcome pairing. We applied AMSTAR‐2 and credibility assessments to provide a clearer comparison of which strategies demonstrate consistent benefits and where evidence remains weak. However, there were some limitations to our study. First, the included systematic reviews had heterogeneities in the study populations, interventions, and outcomes. Definitions (e.g., polypharmacy), criteria (e.g., for PIMs and PPOs), and scales (e.g., for medication adherence and QoL) differed among them. There are apparent differences observed across Table 2, Table 4, and Table S5. However, it does not represent any analytical inconsistency but represents two complementary sets of evaluative criteria. We reported the interpretations made by the original systematic review authors themselves, many of whom based their conclusions on some form of heterogeneity assessment, study design quality, or strength of individual studies. Our credibility grading synthesized data from multiple reviews in an evaluation of broader consistency, direction of effects, and certainty. Because these approaches serve two distinct purposes, some divergence in conclusion is anticipated and represents a methodological feature of umbrella reviews. Second, the number of included primary studies and sample size varied in the systematic reviews, and all the required information was not available from all the systematic reviews. Third, the effectiveness of the interventions over time, contextualizing the societal perspective, was not captured (Table 4).

## 5 Conclusion

Health‐service interventions, particularly pharmacist‐led and multidisciplinary medication reviews and deprescribing interventions, offer the most consistent benefits of medication management in older adults, improving medication appropriateness and reducing inappropriate prescribing, overall medicine burden, falls, and unplanned healthcare use. Other approaches, including education and training, policy and guideline measures such as CDSS and deprescribing guidance, and community‐based counseling or monitoring, showed more modest or variable effects, often limited to adherence or prescribing quality. However, confidence in the overall evidence is constrained by the predominance of low‐quality reviews, substantial methodological heterogeneity, and inconsistent reporting of intervention and outcomes. To advance practice, future research must generate high‐quality, context‐specific evidence using standardized intervention descriptions and outcome measures, especially clinical and economic outcomes.

## Declaration

This work was presented as a poster presentation at the 18th European Public Health Conference in Helsinki, Finland, in November 2025.

## Author Contributions

S.D. and C.S.L. conceptualized the review; S.D. drafted the study protocol; S.D., S.S., and R.A. were involved in the literature search and study selection; S.D., S.S., and R.A. extracted data from the included studies; S.D. performed all qualitative analyses; S.D. and M.B. performed the quantitative analysis related to the overlapping studies; S.D. and D.S. interpreted the analyses; S.D. drafted the review; K.S., J.J.C., A.P., S.P., S.K.T., and C.S.L. provided guidance, expert inputs, and updated the final review. S.D. and D.S. directly accessed and verified the underlying data reported in the manuscript. S.D. is the guarantor and accepts full responsibility for the work and/or the conduct of the study, has access to the data, and controlled the decision to publish.

## Funding

This work was supported by the Department of Health Research, Ministry of Health and Family Welfare, Government of India, through a Long‐Term Fellowship Abroad to SD (File no. R.12023/01/2025‐HR). The open access funding was provided by Karolinska Institutet, Stockholm, Sweden.

## Disclosure

The authors have nothing to report.

## Ethics Statement

The authors have nothing to report.

## Conflicts of Interest

The authors declare no conflicts of interest.

## Supporting information


**Data S1:** Citation matrix: Pairwise overlap matrix of included primary studies across systematic reviews.

**Table S1:** Search strings used in various databases.
**Table S2:** Summarized characteristics of the included systematic reviews.
**Table S3:** Methodological quality assessment of included systematic reviews using the AMSTAR‐2 tool.
**Table S4:** Findings from the included systematic reviews on medication optimization, clinical outcomes, healthcare utilization, economic outcomes, and intervention acceptability.
**Table S5:** Credibility assessment of evidence across intervention outcomes.

## Data Availability

All data related to this work will be available from the corresponding author upon reasonable request.
