# Supplementary material for: Interventions to Optimize Medication Management in Older Adults: An Umbrella Review of Systematic Reviews
Source: J Am Geriatr Soc. 2026 Jun 24;74(8):2297–307. doi: 10.1111/jgs.70561 (PMC13496375; doi:10.1111/jgs.70561)
Supplement: Supplementary file 1 [file JGS-74-2297-s001.pdf]

Supplementary Materials

Table s1. Search strings used in various databases.

**Database**	**Search Terms**
**MEDLINE (via PubMed)**	("Aged"[MeSH] OR older adults OR elderly) AND ("Polypharmacy"[MeSH] OR polypharmacy OR deprescribing OR "Medication Therapy Management"[MeSH] OR "Drug Utilization Review"[MeSH] OR medication review OR prescribing OR "Clinical Decision Support Systems") AND ("Systematic Review"[Publication Type] OR "Meta-Analysis"[Publication Type] OR systematic review OR meta-analysis)
**Embase**	('aged'/exp OR elderly:ti,ab OR "older adults") AND ('polypharmacy'/exp OR 'deprescribing'/exp OR 'drug utilization review'/exp OR medication review:ti,ab) AND ('systematic review'/de OR 'meta-analysis'/de OR systematic review:ti,ab)
**Cochrane CENTRAL**	("older adults" OR elderly OR aged) AND (polypharmacy OR deprescribing OR medication review OR clinical decision support) AND (systematic review OR meta-analysis)
**Scopus**	TITLE-ABS-KEY (elderly OR "older adults" OR aged) AND TITLE-ABS-KEY (polypharmacy OR deprescribing OR medication review OR prescribing) AND TITLE-ABS-KEY ("systematic review" OR "meta-analysis")
**Web of Science**	TS=(elderly OR “older adults” OR aged) AND TS=(polypharmacy OR deprescribing OR medication review OR prescribing) AND TS=(“systematic review” OR “meta-analysis”)

Table s2. Summarized characteristics of the included systematic reviews.

Systematic review (author, year)	Databases included	Number of systematic reviews included	Designs of included systematic reviews	Setting	Number of individuals studied	Population included	Intervention details	Comparator	Primary outcome	Follow-up duration	Reported risk of bias of systematic reviews included
Ali 2020[s1]	MEDLINE, Embase, CINAHL, Cochrane CENTRAL, and Cochrane Library	9	RCTs and observational studies	Community, residential care facility, and outpatient	2,424	Older adults (>65 years) on polypharmacy	Medication review, geriatric assessment/medication screening, and deprescribing	Standard care	Mobility outcomes (fall and functional decline)	Up to 36 months	Moderate to high
Alldred 2016[s2]	Cochrane CENTRAL, MEDLINE, Embase, and CINAHL	12	RCTs and cluster RCTs	Care homes	10,953	Older adults	Medication review, multidisciplinary case-conferencing, healthcare professional education, and CDSS	Standard prescribing practices	Adverse drug event, hospital admission, and mortality	Varied	Moderate to high
Almutairi 2020[s3]	MEDLINE, PubMed, PsycINFO, and Google Scholar	25	RCTs and cluster RCTs	Residential aged-care facility	19,576	Older adults	Medication review, staff education, multi-disciplinary case conferencing, and CDSS	Standard care	Medication appropriateness, hospital admission, and mortality	Up to 12 months	Variable
Bloomfield 2020[s4]	Ovid-MEDLINE, Embase, Cochrane Library, and CINAHL	38	RCTs and cluster RCTs	Community	Exact number not reported	Older adults (>65 years)	Comprehensive medication review, educational initiatives, and computerized CDSS	Standard care or other	Mortality, hospitalization, QoL, and fall	Varied	Low to moderate
Bülow 2023[s5]	Cochrane CENTRAL, MEDLINE, Embase, CINAHL, and ICTRP	25	RCTs	Hospital	15,076	Older adults on polypharmacy	Medication review by healthcare professionals	Standard care	Mortality	1-20 months	Low to moderate
Burgos-Alonso 2024[s6]	Web of Science, PubMed, and Cochrane Library	17	RCTs and quasi-experimental studies	Community and hospital settings	6,463	Older adults (>65 years) on	Counseling, educational program, simplifying regimen, reminder system, monitoring, telephone calls,	Standard care	Medication adherence	Varied	High

						polypharmacy	medication review, and medication package instructions				
Carollo 2024[s7]	PubMed, Embase, and Scopus	30	RCTs, quasi-experimental studies, and cohort studies	Hospitalized older patients	1,808	Frail older adults on polypharmacy	Medication review and deprescribing	Standard care	Medication appropriateness, hospital readmission, duration of hospitalization, emergency visit, QoL, and mortality	Varied	High
Clarkson 2023[s8]	PubMed and Embase	19	RCTs, cluster RCTs, observational studies, feasibility studies, and quality improvement studies	Specialist outpatient clinics	10,914	Older adults	Deprescribing with multidisciplinary team	Standard care	Medication burden	Varied	High
Clyne 2016[s9]	PubMed, Embase, and Cochrane Library	12	RCTs and cluster RCTs	Primary care and community	1,84,015	Community-dwelling older adults	Organizational (medication review), professional (CDSS), financial, regulatory, and multifaceted	Standard prescribing practices	Prescribing of PIMs	Varied	High
Cole 2023[s10]	Cochrane CENTRAL, MEDLINE, Embase, and CINAHL	38	RCTs and cluster RCTs	Hospital, primary care, home-delivered care, and nursing homes	18,073	Older adults on polypharmacy	Complex multidisciplinary (educational program, medication review clinics and prescribing audits, prescribing incentive schemes, and regulatory)	Standard care	Medication appropriateness, PIMs, prescribing omission, and hospital admission	Varied (up to 24 months)	High
Cooper 2015[s11]	MEDLINE, Embase, and Cochrane CENTRAL	12	RCTs, cluster RCTs, and quasi-experimental studies	Hospital, primary care, and nursing homes	22,438	Older adults on polypharmacy	Medication review, patient education, and medication scheduling tools	Standard care	Medication appropriateness and hospitalization	Varied	Moderate to high
Croke 2023[s12]	MEDLINE, CINAHL, and PsycINFO	23	RCTs, cluster RCTs, and quasi-experimental studies	Primary care	23,516	Patients seeking primary care	Pharmacist integration in general practice	Standard care	Prescribing of PIMs and number of prescribed medications	Varied	Moderate to high

Cross 2020[s13]	MEDLINE, Embase, Cochrane CENTRAL, PsycINFO, and CINAHL Plus	50	RCTs, cluster RCTs, and quasi-experimental studies	Community-dwelling older adults	14,269	Older adults on polypharmacy	Educational, behavioral, and mixed aimed at improving medication-taking ability and/or medication adherence	Standard care	Medication adherence, emergency admission, and QoL	Varied	High
Dalton 2018[s14]	MEDLINE, Embase, and Cochrane Library	8	RCTs, interrupted time series, and quasi-experimental studies	Hospital	48,204	Hospitalized older adults	Computerized CDSS	Standard prescribing practices	Reduction in PIMs	Varied	Low to moderate
de Andrade 2024[s15]	LILACS, PubMed, Embase, Cochrane Library, and Web of Science	1	RCT	Palliative care settings	130	Older adults (>75 years) on polypharmacy having limited life expectancy	STOPPFrail tool for deprescribing	Standard care	Reduction in PIMs and medication cost	3 months	High
Dongo 2023[s16]	MEDLINE and ResearchGate	11	RCTs, cluster RCTs, and quasi-experimental studies	Hospital	9,016	Older adults (>60 years)	Pharmacist-initiated strategies	Standard care	Prescription error, reduction in drug-related problem, and safe prescribing	Not specified	Moderate
Earl 2020[s17]	MEDLINE and CINAHL	27	RCTs	Community	88,833	Older adults with multimorbidity	CDSS, educational, and medication review by pharmacists and/or healthcare providers	Standard care	Reduction in polypharmacy and PIMs	Varied	Moderate to high
Faulkner 2022[s18]	CINAHL, Cochrane Library, Embase, MEDLINE, PsycINFO, and Web of Science	1	Quasi-experimental studies	Primary care	54	Frail older adults (>65 years) on polypharmacy having two or more multimorbidity	Pharmacist-led medication review using Beer's and STOPP-START criteria	Standard care	Number of medicines and number of PIMs	Not specified	High
Forsetlund 2011[s19]	Cochrane Library, MEDLINE, Embase, ISI Web of	20	RCTs	Nursing homes	14,826	Nursing home residents	Educational outreach initiatives, educational meetings, medication review, geriatric assessment	Standard care	Reduction in inappropriate drug use	Varied	High

	Knowledge, DARE, and HTA						and care teams, early psychiatric intervention, and activity program for residents				
George 2008[s20]	MEDLINE, Embase, CINAHL, PsycINFO, and International Pharmaceutical Abstracts	8	RCTs	Community	4,140	Older adults on three or more medications	Behavioural (adherence monitoring), educational (by physician, pharmacist, nurse, and others), provider focused (education and medication review)	Standard care	Medication adherence	≥4 weeks	Varied
Gorman 2022[s21]	CINAHL, Cochrane Library, Embase, MEDLINE, PsycINFO, Web of Science, SCOPUS	2	RCT and feasibility study	Primary care	201	Older adults on polypharmacy	Use of validated tools (STOPP-START, MAI, and Beer's Criteria)	Standard prescribing practices	Appropriate polypharmacy	Varied	High
Gutiérrez Valencia 2016[s22]	MEDLINE, Embase, CINAHL, and Cochrane Library	18	RCTs, quasi-experimental studies, and uncontrolled studies	Hospitalized older adults	9,329	Older adults (>65 years)	Medication review, detection of predefined PIMs, counseling, computerized prescription support, and training of nurses	Standard care	Medication appropriateness and clinical outcomes	Varied	High
Hasan Ibrahim 2021[s23]	Ovid-MEDLINE, Embase, Scopus, Web of Science, International Pharmaceutical Abstracts, CINAHL, Cochrane CENTRAL, and Cochrane Library	7	RCTs, quasi-experimental studies, and controlled pre-and-post studies	Primary care and general practitioner	16,854	Older adults on and polypharmacy having multimorbidity	Pharmacist-led medication review accompanied by recommendations agreed and implemented by general practitioners	Standard care	Medicine-related problems, prescription appropriateness, and medication adherence	Varied	Moderate to high
Hatah 2014[s24]	MEDLINE, Embase, and International Pharmaceutical Abstracts	36	RCTs, cluster RCTs, controlled pre-and-post studies, and cohort studies	Pharmacy, patient's home, community health center, and general practitioner clinics	Exact number not reported	Pharmacist-led medication review	Pharmacist-led medication review	Standard care	Changes in clinical biomarkers of common diseases or, hospitalization, and mortality	Varied	Moderate to high

Hikaka 2019[s25]	Ovid-MEDLINE, Embase, and Scopus	7	Prospective and retrospective case studies, semi-structured interviews	Outpatient settings	542	Community-dwelling older adults	Pharmacist-led medication review	Standard care	Drug-related problems, medication adherence, and knowledge about medicines	Not specified	Not clear
Hill-Taylor 2016[s26]	PubMed, Embase, CINAHL, and Web of Science	4	RCTs	Acute and long-term care	1,925	Older adults	STOPP-START criteria implementation	Standard care	Proportion of PIM rate and clinical outcomes	Varied	Moderate to high
Huiskes 2017[s27]	MEDLINE, Embase, and Web of Science	31	RCTs	General healthcare	15,509	No restrictions	Medication review	Standard care	Clinical outcomes, drug-related outcomes	Varied	Mostly low
Ibrahim 2021[s28]	MEDLINE, Embase, CINAHL, PsycINFO, Web of Science, and Cochrane Library	6	RCTs, pre and post comparison studies, and cohort studies	Primary care, Hospital, Residential care facility, Community setting	657	Older adults with frailty	Pharmacist/ multidisciplinary team-led deprescribing	Standard care	Safety of deprescribing	Varied	Moderate
Johansson 2016[s29]	MEDLINE, Embase, and Cochrane Library	25	RCTs, Cluster RCTs, quasi-experimental studies, and case-control studies	Primary care, hospital care, and nursing homes	10,980	Older adults on polypharmacy	Pharmacist-led, physician-led, multidisciplinary team-led review, CDSS, use of valid tools (PIMs, MAI, and STOPP-START criteria)	Standard care	Mortality, hospitalization, and change in number of medicines	Varied	Moderate to high
Kim 2024[s30]	PubMed, Embase, Cochrane CENTRAL, KMbase, and RISS	10	RCTs and quasi-experimental studies	Healthcare settings (outpatient, inpatient, and nursing homes)	2,163	Older adults aged >65 years residing in Asian Countries	Pharmacist-led medication review	Standard care	Hospitalization, mortality, and QoL	Varied	Moderate to high
Kua 2019[s31]	MEDLINE, Embase, CINAHL, Cochrane Library, and International	41	RCTs	Nursing homes	18,408	Residents aged >60 years	Drug discontinuation, medication review, educational training, and others	Standard care	Fall, mortality, hospitalization, and PIMs	Varied	Low to moderate

	Pharmaceutical Abstracts										
Laberge 2021[s32]	Ovid-MEDLINE, Embase, CINAHL, AgeLine, Cochrane Library, Web of Science	11	RCTs, cluster RCTs, pre-and-post studies, and cohort studies	Primary care clinics, Nursing homes, Pharmacies, hospital, academic medical center, clinical sites	6,375	Older adults on polypharmacy having multimorbidity	Pharmacist or research physician-led medication review, CDSS, and pharmacogenomic testing	Standard care	Cost-benefit analysis	Varied	High
Lee 2021[s33]	MEDLINE, Embase, Cochrane CENTRAL, and CINAHL	5	RCTs	Community, nursing home, and emergency department	1,305	Older adults aged >65 years	Deprescribing fall-risk increasing drugs	Standard care	Rate and incidence of fall	6-12 months	Moderate to high
Liu 2024[s34]	MEDLINE, Embase, Cochrane Library, Cochrane CENTRAL, PsycINFO, ANZCTR, and ICTRP	9	RCTs	Community, nursing homes, and hospitals	5,720	Older adults	Pharmacist/ geriatrician-led deprescribing (medication review)	Standard care	Reduction in the drug burden index	3-12 months	High
Loganathan 2011[s35]	MEDLINE, EMBASE, International Pharmaceutical Abstracts, and Cochrane Library	16	RCTs, cluster RCTs, and controlled pre-and-post studies	Nursing homes, residential homes, long-term care facility, and mixed homes	11,534	Older adults	Staff education, MDT meetings, pharmacist-led medication review, and computerized CDSS	Standard prescribing practices	Appropriate prescribing	3 months to 5 years	
Maeda 2009[s36]	MEDLINE and Cochrane Library	27 articles, including 28	Controlled clinical trials, RCTs, and cluster RCTs	Nursing homes, hospitals, and	Exact number not reported	Older adults on polypharmacy	Medication review by pharmacists	Standard care	Reduction in the number of medications	1 month to 2.5 years	Moderate

		controlled studies		outpatient clinics							
Masnoon 2024[s37]	MEDLINE, Embase, Scopus, and CINAHL	17	RCTs and quasi-experimental studies	Community, nursing home, and care facility	8,310	Older adults aged >60 years	Medication review	Standard care	Number of medications, medication appropriateness, and hospital readmission	Varied	Low to moderate
Mizokami 2019[s38]	MEDLINE and Google Scholar	9	RCTs	Hospital	5,299	Older adults on polypharmacy	Prescription review, adherence review, or comprehensive clinical evaluation for disease management	Standard care	Unplanned hospitalization and re-hospitalization	3 to 12 months	Moderate to high
Monteiro 2019[s39]	MEDLINE, Embase, Cochrane CENTRAL, and Web of Science	16	RCTs, quasi-experimental studies, and crossover studies	Primary and secondary care	2,66,562	Older adults aged >65 years	Computerized CDSS	Standard care	Reduction in PIMs	Varied	Moderate
Mucherino 2022[s40]	PubMed and EMBASE	20	Cross-sectional studies, cohort studies, cluster RCTs, quasi-experimental studies, and interrupted time-series	Hospital, nursing homes, community, and geriatric units	7,70,63,962	Older adults on PIMs	Explicit criteria for identifying medication appropriateness (Beer's criteria, PRISCUS list, or STOPP-START criteria)	Standard care	Cost variation, effectiveness, and avoidable healthcare costs	Varied	Moderate
Nakham 2020[s41]	CINAHL, MEDLINE, Embase, and Cochrane CENTRAL	8	RCTs and quasi-experimental studies	Hospitals, community, nursing homes, and retirement villages	1,061	Older adults aged >65 with anticholinergic burden	Pharmacist-led patient medication review followed by feedback to the prescribers	Standard care	Reduction in anticholinergic burden	Varied	Moderate
Njoku 2024[s42]	MEDLINE, Scopus, Ovid-Emare, Cochrane CENTRAL, Web of Science, and Google Scholar	3	RCT and clustered RCT	Residential aged care (nursing homes and long-term care)	1,030	Older adults with frailty	Medication optimization delivered with exercise or physical activity and/or nutritional support/education interventions	Standard care or passive intervention	Fragility, number of medications, nutritional status, and cost	3-9 months	Low to serious

Omuya 2023[s43]	Medline, PubMed, Scopus, Cochrane Library, and Web of Science	14	RCTs	Nursing homes, hospitals, primary care, and community pharmacies	8,913	Older adults on polypharmacy	Deprescribing (comprehensive medication review)	Standard care	Number or dose of medications, health-related QoL, cost, mortality, hospitalization, emergency visits, and fall	4-12 months	Moderate to high
O'Shea 2022[s44]	PubMed, Embase, Cochrane CENTRAL, CINAHL, AMED, and PsycINFO	15	Case series, cohort studies, case-control studies, and RCTs	Primary care and hospitals	3,899	Older adults on polypharmacy having with multimorbidity	Pharmacogenetic-based medication review	Standard care	Mortality, mental health, medication appropriateness, serious adverse drug reaction, medication regimen complexity, adverse effects, health-related QoL, and fall	Varied	Moderate to high
Page 2016[s45]	CINAHL Plus, Ovid-MEDLINE, Scopus, Web of Science, Embase, and ProQuest	132	RCTs, quasi-experimental studies, n quasi-experimental studies, and observational studies	Hospital, residential aged care facility, community (outpatient facility, general practice, and retirement villages)	34,143	Older adults aged >65 years on one or more regular medications	Deprescribing by health care professional	Standard care or no comparator	Mortality	Not specified	Uncertain or high
Patterson 2014[s46]	MEDLINE, Embase, CINAHL, PsycINFO, and EBMR	12	RCTs, cluster RCTs, and quasi-experimental studies	Community, nursing home, and hospital	Exact number not reported	Older adults on polypharmacy	Pharmacist-led medication review, co-ordinated transition from hospital to long-term care facility, pharmacist consultations with patients and physicians, and CDSS	Standard care	Medication appropriateness, PIMs, and hospital admission	Varied	Moderate to low

Pazan 2021[s47]	MEDLINE	25	RCTs	Geriatric healthcare	4,954	Older adults with frailty	Medication optimization including medication review, education, care coordination, clinical decision support system technology, and brown bag analyses	Standard care	Frailty	Not specif ied	Low to moderate
Pazan 2022[s48]	MEDLINE and Web of Science	13	RCTs	Nursing homes, hospital, and community	3,657	Older adults (aged ≥65 years) having multimorbi dity	Medication optimization including medication review, education, care coordination, computerized CDSS, and brown bag analyses	Standard care	Cognitive function	Not specif ied	Moderate
Quek 2024[s49]	CINAHL, MEDLINE, Embase, Scopus, Web of Science, and ProQuest	259	RCTs, cluster RCTs, quasi-experimental studies, retrospective cohort, prospective cohort, and case-control studies	Communit y, hospital, and residential aged care facility settings	3,03,556	Older adults (≥65 years) on at least one regular medication	Deprescribing, practitioner education, and computerized CDSS	Standard care	Mortality	Varie d	Moderate to high
Rao 2024[s50]	MEDLINE	7	RCTs and observational studies	Communit y and hospital	Exact number not reported	Older adults on PIM	Medication therapy management	Standard care	Reduction in PIMs	<4 mont hs	Moderate
Reeve 2022[s51]	MEDLINE, Embase, CINAHL, Cochrane Library, Cochrane CENTRAL, Google, Google Scholar, and JBISRIR	32	RCTs, quasi-experimental studies, interrupted time series, observational studies (cohort, case-control, cross-sectional, case series). and qualitative studies (interviews)	Primary, secondary, and tertiary care settings and pharmacy call center	Exact number not reported	Older adults aged >65 years on polypharma cy having multimorbi dity	Multidisciplinary and shared deprescribing	Standard care	Medication reduction, clinical outcome, safety, and acceptability	Not specif ied	Moderate .
Rodrigu es 2022[s52]	MEDLINE, PubMed, EMBASE, and Cochrane Library	47	RCT, case series, quasi-experimental studies, cohort study, retrospective study, and interrupted time	Hospital and p primary care	Exact number not reported	Older adults aged ≥65 years	Medication review, educational strategy, CDSS, and organizational and multifaceted approaches	Standard care	PIM reduction	Varie d	Moderate to high

			series								
Romano 2022[s53]	MEDLINE, EconLit, Scopus, Web of Science, Tuft University-Cost Effectiveness Analysis, York University-Centre for Reviews and Dissemination, and Google Scholar	14	RCTs, cluster RCTs, interrupted time series, quasi-experimental studies, and observational studies	Communit y and primary care	8,743	Adults aged ≥65 years	Medication review with or without a supportive educational component delivered within a pharmacist-physician collaboration	Standard care	Cost-effectiveness, QALY, number of falls, and medication appropriateness	2-12 mont hs	Low to high
Roncal-Belzunc e 2024[s54]	MEDLINE, Embase, and Cochrane CENTRAL	17	RCTs	Primary care, outpatient clinics, and community pharmacies	Exact number not reported	Older adults on polypharma cy	Multidisciplinary medication review	Standard care	Change in functional and cognitive status	Varie d	High
Saeed 2022[s55]	MEDLINE, Scopus, Embase, Web of Science, International Pharmaceutical Abstracts, CINAHL Plus, Cochrane Library, Cochrane CENTRAL, Clinicaltrials.gov, ICTRP, and Research Registry	3	RCTs and quasi-experimental studies	Secondary or acute care facility	1,133	Frail older adults	Multidisciplinary team- or physician-led deprescribing	Standard care	Medication appropriateness, change in medication, adverse drug reaction, mortality, QoL, fall, fracture, disability, and cost of medication and or healthcare utilization	Not specif ied	Moderate to high
Seppala 2022[s56]	MEDLINE, Embase, Cochrane CENTRAL, and PsycINFO	49	RCTs and observational studies	Communit y, hospital, and long-term care setting	Exact number not reported	Older adults aged >65 years	Deprescribing including pharmacist- or physician-led medication review, education programs for prescribers, and CDSS	Standard care	Medication appropriateness, mortality, and fall	Varie d	Moderate
Shafiee Hanjani 2019[s57]	Cochrane Library, MEDLINE, Embase, PsycINFO, and CINAHL	18	RCTs, quasi-experimental studies, quasi-experimental studies, and observational studies	Nursing homes, hospital, and	3,047	Older adults with dementia	Single profession- or multi-disciplinary team-led medication optimization (medication review, deprescribing, minimizing	Standard care	Medication appropriateness	Not specif ied	Low to moderate

				community settings			adverse drug reactions, geriatric assessment and consultation, interdisciplinary geriatric and psychiatric care education, and computerized CDSS)				
Shrestha 2020[s58]	PubMed, Embase, CINAHL, PsycINFO, and Google Scholar	9	RCTs, quasi-experimental studies, quasi-experimental studies, and observational studies	Healthcare settings	1,375	Older adults having life-limiting illness and limited life expectancy	Deprescribing	Standard care	Medication appropriateness	12-24 months	Moderate.
Shrestha 2021[s59]	MEDLINE, Embase, CINAHL, PsycINFO, and Google Scholar	5	RCTs and quasi-experimental studies	Hospital and residential aged care facility	689	Older adults at end-of-life, on with least one life-limiting illness and on at least one PIM	Deprescribing and comprehensive medication review	Standard care	QoL, physical function, cognitive function scores, mortality, fall, non-vertebral fracture, unplanned hospital admission, emergency presentation, general practitioner visit, and depression	Varied	Moderate to low
Sirois 2024[s60]	PubMed and Cochrane Library	12	RCTs	Community-dwelling older adults	Exact number not reported	Older adults on polypharmacy	Deprescribing	Standard care	Mortality	≤1 year	High
Tasai 2021[s61]	PubMed, EMBASE, Cochrane CENTRAL, and International Pharmaceutical Abstracts	4	RCTs	Community pharmacy	4,633	Older adults aged ≥65 years on polypharmacy	Community pharmacist-led medication review	Standard care	Hospitalization, emergency visits, QoL, or medication adherence	Not specified	Low to moderate

Tecklenborg 2020[s62]	Ovid-MEDLINE, Embase, Informit, International Pharmaceutical Abstracts, Scopus, Cochrane CENTRAL, CINAHL, and PsycINFO	9	RCTs	Hospitalized older patients	2,522	Older adults (≥65 years)	Pharmacist-, or physician-, or multidisciplinary team-led deprescribing	Standard care	Reduction in PIMs	Varied	High
Thillainadesan 2018[s63]	MEDLINE, Embase, CINAHL, Web of Science, Cochrane Library, and Cochrane CENTRAL	7	RCTs and case control studies	Community (primary) care	Exact number not reported	Older adults aged ≥65 years	Medication review by pharmacist/primary care physician/research team, and education	Standard care	Reduction in adverse drug reactions or associated health outcomes (hospitalization, emergency department visit, mortality, quality-of-life, mental health, and physical function)	Varied	Moderate to high
Thomas 2014[s64]	MEDLINE, EMBASE, and Cochrane Library	20	RCTs	Hospital and community	9,858	Older adults aged ≥60 years	Hospital or community pharmacist-led medication review, education and counseling to patients	Standard care	Unplanned hospital admission	Varied	Moderate
Tjia 2013[s65]	MEDLINE, EMBASE, and IPA	36	RCTs, quasi-experimental studies, and case series	Nursing homes and hospitals	13,906	Frail older adults aged ≥65 years	Deprescribing	Standard care	Reduction in unnecessary medications	Not specified	Low to moderate
Ulley 2019[s66]	ASSIA, Cochrane CENTRAL, CINAHL, MEDLINE, PsycINFO, Scopus, Sociological Abstracts, and Web of Science	22	RCTs, observational studies, and cohort studies	Community-dwelling older adults	Exact number not reported	Older adults on polypharmacy	Pharmacist-led and general practitioner-led deprescribing	Standard care	Medication adherence	6-12 months	Moderate
Vaismoradi 2024[s67]	PubMed, Scopus, CINAHL, ProQuest, and Embase	32	RCTs, quasi-experimental studies, qualitative studies,	Primary care and	Exact number	Older adults aged >65 on	Nurse-led medication optimization programs	Standard care	Medication adherence and	Not specified	Low to moderate

			mixed method studies, and cross-sectional studies	long-term care	not reported	polypharmacy			healthcare utilization		
Walsh 2016[s68]	MEDLINE, PubMed, EMBASE, Cochrane Library, Web of Science, and ClinicalTrials.gov	4	RCTs and quasi-experimental studies	Hospitalized older patients	1,164	Older adults aged ≥65 years	Pharmacist-led use of validated tools (MAI and Beers criteria)	Standard care	Reduction in PIM	Not specific	Moderate
Williams 2008[s69]	CINAHL, MEDLINE, PubMed, and Web of Science	8	RCTs	Community-dwelling older adults	Exact number not reported	Older adults having multiple multimorbidity	Pharmacist- and nurse-led medication adherence programs	Standard care	Medication adherence	≥3 months	High
Zhou 2023[s70]	PubMed, EMBASE, and Cochrane Library	32	RCTs	Hospital, nursing homes, and primary care	18,670	Older adults aged ≥60 years	Multidisciplinary deprescribing	Routine care	Reduction in PIMs	Varied	Low
Zhou 2023[s71]	Cochrane Library, PubMed, EMBASE, Web of Science, and ClinicalTrials.gov	14	RCTs	Hospital and community settings	65,971	Older adults aged ≥65 years	Medication review and optimization by pharmacists, physicians, and CDSS	Standard care	Reduction in PIMs and hospital readmission	Not specified	Moderate

AMED: Allied and Complementary Medicine Database, ASSIA: Applied Social Sciences Index and Abstracts, CDSS: clinical decision support system, DARE: Database of Abstracts of Reviews of Effects, EMBR: Evidence-Based Medicine Reviews, HTA: Health Technology Assessment, ICTRP: International Clinical Trials Registry Platform, JBISRIR: Joanna Briggs Institute Database of Systematic Reviews and Implementation Reports, KMbase: Korean Medicine Database, MAI: medication appropriateness index, PIMs: potentially inappropriate medications, QALY: quality-adjusted life year, QoL: quality of life, RCT: randomized controlled trial, RISS: Research Information Sharing Service, STOPP-START: Screening Tool of Older Persons' Prescriptions and Screening Tool to Alert to Right Treatment.

Table s3. Methodological quality assessment of each included systematic review using the AMSTAR-2 (A MeaSurement Tool to Assess systematic Reviews) tool.

**Systematic review (author, year)**	**Did the research questions and inclusion criteria include the components of PICO?**	**Did the review methods get established prior to the conduct of the review?**	**Did the authors explain their selection of study designs for inclusion?**	**Was a comprehensive literature search strategy used?**	**Was study selection performed in duplicate?**	**Was data extraction performed in duplicate?**	**Did the authors provide a list of excluded studies with justifications?**	**Were the included studies described in adequate detail?**	**Was a satisfactory technique used for assessing the risk of bias (RoB)?**	**Did the authors report on the sources of funding for included studies?**	**If meta-analysis was performed, were appropriate methods used?**	**If meta-analysis was performed, was the potential impact of RoB assessed?**	**Did the authors account for RoB in primary studies in the discussion?**	**Was heterogeneity in results discussed satisfactorily?**	**If quantitative synthesis was performed, was publication bias investigated?**	**Did the authors report potential conflicts of interest?**
Ali 2020[s1]	Yes	Yes	Yes	Yes	Yes	Yes	Yes	Yes	Yes	Yes	Yes	Yes	Yes	Yes	Yes	Yes
Alldred 2016[s2]	Partial	Partial	Partial	Partial	Partial	Partial	Partial	Partial	Partial	Partial	Partial	Partial	Partial	Partial	Partial	Partial
Almutairi 2020[s3]	No	No	No	No	No	No	No	No	No	No	No	No	No	No	No	No
Bloomfield 2020[s4]	Yes	Yes	Yes	Yes	Yes	Yes	Yes	Yes	Yes	Yes	Yes	Yes	Yes	Yes	Yes	Yes
Bülow 2023[s5]	Partial	Partial	Partial	Partial	Partial	Partial	Partial	Partial	Partial	Partial	Partial	Partial	Partial	Partial	Partial	Partial
Burgos-Alonso 2024[s6]	No	No	No	No	No	No	No	No	No	No	No	No	No	No	No	No
Carollo 2024[s7]	Yes	Yes	Yes	Yes	Yes	Yes	Yes	Yes	Yes	Yes	Yes	Yes	Yes	Yes	Yes	Yes
Clarkson 2023[s8]	Partial	Partial	Partial	Partial	Partial	Partial	Partial	Partial	Partial	Partial	Partial	Partial	Partial	Partial	Partial	Partial
Clyne 2016[s9]	No	No	No	No	No	No	No	No	No	No	No	No	No	No	No	No
Cole 2023[s10]	Yes	Yes	Yes	Yes	Yes	Yes	Yes	Yes	Yes	Yes	Yes	Yes	Yes	Yes	Yes	Yes
Cooper 2015[s11]	Partial	Partial	Partial	Partial	Partial	Partial	Partial	Partial	Partial	Partial	Partial	Partial	Partial	Partial	Partial	Partial
Croke 2023[s12]	No	No	No	No	No	No	No	No	No	No	No	No	No	No	No	No
Cross 2020[s13]	Yes	Yes	Yes	Yes	Yes	Yes	Yes	Yes	Yes	Yes	Yes	Yes	Yes	Yes	Yes	Yes
Dalton 2018[s14]	Partial	Partial	Partial	Partial	Partial	Partial	Partial	Partial	Partial	Partial	Partial	Partial	Partial	Partial	Partial	Partial
de Andrade 2024[s15]	No	No	No	No	No	No	No	No	No	No	No	No	No	No	No	No
Dongo 2023[s16]	Yes	Yes	Yes	Yes	Yes	Yes	Yes	Yes	Yes	Yes	Yes	Yes	Yes	Yes	Yes	Yes
Earl 2020[s17]	Partial	Partial	Partial	Partial	Partial	Partial	Partial	Partial	Partial	Partial	Partial	Partial	Partial	Partial	Partial	Partial
Faulkner 2022[s18]	No	No	No	No	No	No	No	No	No	No	No	No	No	No	No	No

Forsetlund 2011[s19]	Yes	Yes	Yes	Yes	Yes	Yes	Yes	Yes	Yes	Yes	Yes	Yes	Yes	Yes	Yes	Yes
George 2008[s20]	No	No	No	No	No	No	No	No	No	No	No	No	No	No	No	No
Gorman 2022[s21]	Partial	Partial	Partial	Partial	Partial	Partial	Partial	Partial	Partial	Partial	Partial	Partial	Partial	Partial	Partial	Partial
Gutiérrez Valencia 2016[s22]	Yes	Yes	Yes	Yes	Yes	Yes	Yes	Yes	Yes	Yes	Yes	Yes	Yes	Yes	Yes	Yes
Hasan Ibrahim 2021[s23]	Partial	Partial	Partial	Partial	Partial	Partial	Partial	Partial	Partial	Partial	Partial	Partial	Partial	Partial	Partial	Partial
Hatah 2014[s24]	No	No	No	No	No	No	No	No	No	No	No	No	No	No	No	No
Hikaka 2019[s25]	Yes	Yes	Yes	Yes	Yes	Yes	Yes	Yes	Yes	Yes	Yes	Yes	Yes	Yes	Yes	Yes
Hill-Taylor 2016[s26]	Partial	Partial	Partial	Partial	Partial	Partial	Partial	Partial	Partial	Partial	Partial	Partial	Partial	Partial	Partial	Partial
Huiskes 2017[s27]	Yes	Yes	Yes	Yes	Yes	Yes	Yes	Yes	Yes	Yes	Yes	Yes	Yes	Yes	Yes	Yes
Ibrahim 2021[s28]	Partial	Partial	Partial	Partial	Partial	Partial	Partial	Partial	Partial	Partial	Partial	Partial	Partial	Partial	Partial	Partial
Johansson 2016[s29]	No	No	No	No	No	No	No	No	No	No	No	No	No	No	No	No
Kim 2024[s30]	Yes	Yes	Yes	Yes	Yes	Yes	Yes	Yes	Yes	Yes	Yes	Yes	Yes	Yes	Yes	Yes
Kua 2019[s31]	Yes	Yes	Yes	Yes	Yes	Yes	Yes	Yes	Yes	Yes	Yes	Yes	Yes	Yes	Yes	Yes
Laberge 2021[s32]	No	No	No	No	No	No	No	No	No	No	No	No	No	No	No	No
Lee 2021[s33]	Yes	Yes	Yes	Yes	Yes	Yes	Yes	Yes	Yes	Yes	Yes	Yes	Yes	Yes	Yes	Yes
Liu 2024[s34]	Yes	Yes	Yes	Yes	Yes	Yes	Yes	Yes	Yes	Yes	Yes	Yes	Yes	Yes	Yes	Yes
Loganathan 2011[s35]	Partial	Partial	Partial	Partial	Partial	Partial	Partial	Partial	Partial	Partial	Partial	Partial	Partial	Partial	Partial	Partial
Maeda 2009[s36]	No	No	No	No	No	No	No	No	No	No	No	No	No	No	No	No
Masnoon 2024[s37]	Partial	Partial	Partial	Partial	Partial	Partial	Partial	Partial	Partial	Partial	Partial	Partial	Partial	Partial	Partial	Partial
Mizokami 2019[s38]	Yes	Yes	Yes	Yes	Yes	Yes	Yes	Yes	Yes	Yes	Yes	Yes	Yes	Yes	Yes	Yes
Monteiro 2019[s39]	Partial	Partial	Partial	Partial	Partial	Partial	Partial	Partial	Partial	Partial	Partial	Partial	Partial	Partial	Partial	Partial
Mucherino 2022[s40]	No	No	No	No	No	No	No	No	No	No	No	No	No	No	No	No
Nakham 2020[s41]	Yes	Yes	Yes	Yes	Yes	Yes	Yes	Yes	Yes	Yes	Yes	Yes	Yes	Yes	Yes	Yes
Njoku 2024[s42]	Yes	Yes	Yes	Yes	Yes	Yes	Yes	Yes	Yes	Yes	Yes	Yes	Yes	Yes	Yes	Yes
Omuya 2023[s43]	Partial	Partial	Partial	Partial	Partial	Partial	Partial	Partial	Partial	Partial	Partial	Partial	Partial	Partial	Partial	Partial
O'Shea 2022[s44]	No	No	No	No	No	No	No	No	No	No	No	No	No	No	No	No
Page 2016[s45]	Yes	Yes	Yes	Yes	Yes	Yes	Yes	Yes	Yes	Yes	Yes	Yes	Yes	Yes	Yes	Yes
Patterson 2014[s46]	Partial	Partial	Partial	Partial	Partial	Partial	Partial	Partial	Partial	Partial	Partial	Partial	Partial	Partial	Partial	Partial
Pazan 2021[s47]	Yes	Yes	Yes	Yes	Yes	Yes	Yes	Yes	Yes	Yes	Yes	Yes	Yes	Yes	Yes	Yes

Pazan 2022[s48]	Partial	Partial	Partial	Partial	Partial	Partial	Partial	Partial	Partial	Partial	Partial	Partial	Partial	Partial	Partial	Partial
Quek 2024[s49]	No	No	No	No	No	No	No	No	No	No	No	No	No	No	No	No
Rao 2024[s50]	Yes	Yes	Yes	Yes	Yes	Yes	Yes	Yes	Yes	Yes	Yes	Yes	Yes	Yes	Yes	Yes
Reeve 2022[s51]	Partial	Partial	Partial	Partial	Partial	Partial	Partial	Partial	Partial	Partial	Partial	Partial	Partial	Partial	Partial	Partial
Rodrigues 2022[s52]	Yes	Yes	Yes	Yes	Yes	Yes	Yes	Yes	Yes	Yes	Yes	Yes	Yes	Yes	Yes	Yes
Romano 2022[s53]	Partial	Partial	Partial	Partial	Partial	Partial	Partial	Partial	Partial	Partial	Partial	Partial	Partial	Partial	Partial	Partial
Roncal-Belzunce 2024[s54]	No	No	No	No	No	No	No	No	No	No	No	No	No	No	No	No
Saeed 2022[s55]	Yes	Yes	Yes	Yes	Yes	Yes	Yes	Yes	Yes	Yes	Yes	Yes	Yes	Yes	Yes	Yes
Seppala 2022[s56]	Yes	Yes	Yes	Yes	Yes	Yes	Yes	Yes	Yes	Yes	Yes	Yes	Yes	Yes	Yes	Yes
Shafiee Hanjani 2019[s57]	Partial	Partial	Partial	Partial	Partial	Partial	Partial	Partial	Partial	Partial	Partial	Partial	Partial	Partial	Partial	Partial
Shrestha 2020[s58]	No	No	No	No	No	No	No	No	No	No	No	No	No	No	No	No
Shrestha 2021[s59]	Yes	Yes	Yes	Yes	Yes	Yes	Yes	Yes	Yes	Yes	Yes	Yes	Yes	Yes	Yes	Yes
Sirois 2024[s60]	Partial	Partial	Partial	Partial	Partial	Partial	Partial	Partial	Partial	Partial	Partial	Partial	Partial	Partial	Partial	Partial
Tasai 2021[s61]	Yes	Partial	Yes	Yes	Yes	Partial	No	Yes	Yes	Yes	No	Partial	Yes	Partial	Yes	No
Tecklenborg 2020[s62]	Yes	Yes	Yes	Yes	Yes	Yes	Yes	Yes	Yes	Yes	Yes	Yes	Yes	Yes	Yes	Yes
Thillainadesan 2018[s63]	Yes	Yes	Partial	Yes	Partial	No	Yes	Yes	Partial	Yes	Partial	No	Partial	Yes	No	Yes
Thomas 2014[s64]	Yes	Partial	Yes	Yes	Yes	Partial	No	Yes	Yes	Yes	No	Partial	Yes	Partial	Yes	No
Tjia 2013[s65]	Yes	Yes	Yes	Yes	Yes	Yes	Yes	Yes	Yes	Yes	Yes	Yes	Yes	Yes	Yes	Yes
Ulley 2019[s66]	Yes	Partial	Yes	Yes	Yes	Partial	No	Yes	Yes	Yes	No	Partial	Yes	Partial	Yes	No
Vaismoradi 2024[s67]	Yes	Yes	Yes	Yes	Yes	Yes	Yes	Yes	Yes	Yes	Yes	Yes	Yes	Yes	Yes	Yes
Walsh 2016[s68]	Yes	Partial	Yes	Yes	Yes	Partial	No	Yes	Yes	Yes	No	Partial	Yes	Partial	Yes	No
Williams 2008[s69]	Yes	Yes	Yes	Yes	Yes	Yes	Yes	Yes	Yes	Yes	Yes	Yes	Yes	Yes	Yes	Yes
Zhou 2023[s70]	Yes	Yes	Partial	Yes	Partial	No	Yes	Yes	Partial	Yes	Partial	No	Partial	Yes	No	Yes
Zhou 2023[s71]	Yes	Partial	Yes	Yes	Yes	Partial	No	Yes	Yes	Yes	No	Partial	Yes	Partial	Yes	No

Table s4. Findings from the included systematic reviews on medication optimization, clinical outcomes, healthcare use and economic outcomes, and acceptability of the intervention.

Systematic review (author, year)	Number of medications	Potentially inappropriate medications	Potential prescribing omissions	Medication appropriateness	Adverse drug reactions or drug-related problems	Medication adherence	All-cause mortality	Quality-of-life	Physical functions	Falls	Cognitive function	Duration of hospitalization	Hospital visits, admissions, and re-admissions	Healthcare utilization	Cost	Acceptability of the intervention among physicians	Acceptability of the intervention among patients
Ali 2020[s1]					Uncertain evidence				No significant difference [SMD: -0.00 (-0.21 to 0.20), I^2: 0%]	No significant difference [RR: 0.87 (0.57 to 1.31), I^2: 80%]							
Alldred 2016[s2]				Improvement	Improvement (identification and resolution)		No reduction	Uncertain evidence					Uncertain evidence		Uncertain evidence		
Almutairi 2020[s3]				Significant improvement [RR: 0.71 (0.60 to 0.84), I^2: 91%]	No significant difference [RR: 1.04 (0.96 to 1.13), I^2: 0%]		No significant difference [RR: 0.98 (0.86 to 1.11), I^2: 43%]	No significant difference [SMD: 0.16 (-0.13 to 0.45), I^2: 57%]		No significant difference [RR: 1.06 (0.89 to 1.26), I^2: 87%]	No significant difference [WMD: 0.69 (-1.25 to 2.64), I^2: 0%]		No significant difference [RR: 1.00 (0.93 to 1.06), I^2: 0%]				

Bloomfield 2020[s4]		Very small difference					Significant reduction [OR: 0.74 (0.58 to 0.95), I^2: 0]	Little to No significant difference		Little to no significant difference			No significant difference [ARD: 0.6 (-2.3 to 3.5)]				
Bülow 2023[s5]					Uncertain evidence		No significant difference [RR: 0.96 (0.87 to 1.05), I^2: 0%]	No significant difference [SMD: 0.10 (-0.10 to 0.30), I^2: 0%]					Significant reduction [RR: 0.93 (0.89 to 0.98), I^2: 0%]				
Burgos-Alonso 2024[s6]						Significant improvement [OR: 1.90 (1.10 to 3.27), I^2: 78%]											
Carollo 2024[s7]				54.5% showed significant benefits			No significant difference [HR: 0.98 (0.96 to 1.00), I^2: 0%]						Significant reduction [HR: 0.92 (0.85 to 0.99), I^2: 0%]				

Clarkson 2023[s8]	Significant reductions				Uncertain evidence			Uncertain evidence									
Clyne 2016[s9]		Effective in decreasing new but not existing ones						No significant difference						Uncertain evidence	No significant difference		
Cole 2023[s10]		Significant reductions [SMD: -0.19 (-0.34 to -0.05), I^2: 67%]	No significant reductions [SMD: -0.48 (-1.05 to 0.09), I^2: 92%]	Significant benefit [MD: -5.66 (-9.26 to -2.06), I^2: 97%;	Uncertain evidence	No significant difference		Uncertain evidence					Little or no significant difference				
Cooper 2015[s11]		No evidence [MD: -0.1 (-0.28 to 0.09), I^2: 89%]	Higher reduction	Significant benefit [MD: -3.88 (-5.40 to -2.35), I^2: 0%]				No significant difference					No significant difference	Uncertain evidence			
Croke 2023[s12]	Significant benefit [MD: -0.80 (-1.17 to -0.43), I^2: 57%]	Significant benefit			Favoured pharmacist integration		Uncertain evidence	Uncertain evidence					Reduction in hospitalizations	No significant difference	Medication review and follow-up service was cost-effective		

Cross 2020[s13]						Little or no benefit [RR: 1.22 (1.08 to 1.37), I^2: 77%; SMD: 0.47 (-0.08 to 1.02), I^2: 95%]	No significant difference [RR: 0.93 (0.67 to 1.30), I^2: 33%]	Little or no difference					Reduction [RR: 0.67 (0.50 to 0.90), (I^2: 73%]				
Dalton 2018[s14]		Reduction [ARR: 2 to 5.9%; RRR: 14 to 77.6%; OR: 0.6 (0.38 to 0.93), I^2: 82%]			Uncertain evidence		Uncertain evidence			Uncertain evidence		Uncertain evidence	Uncertain evidence				Uncertain evidence
de Andrade 2024[s15]	Reduction						No significant difference [RR: 0.67 (0.35 to 1.27)]	No significant difference		No significant difference			No significant difference		No significant difference		
Dongo 2023[s16]					Reduction	Uncertain evidence	No difference	Improved					No significant difference		Uncertain evidence		Median acceptance rate: 80%
Earl 2020[s17]	Significant reduction			Significant reduction	Significant reduction		No significant	No significant		Significant reduction	No significant		Significant reduction	No significant	Significant reduction		

							difference	difference			difference			difference			
Faulkner 2022[s18]	No significant reduction	Significant reduction															
Forsetlund 2011[s19]	Significant reduction	Small reduction		Reduction			Reduction			No significant difference [WMD: 2.6% (-18.7 to, 23.8%)]			No significant difference				
George 2008[s20]						No difference [relative change: -13 to 55.5%]											
Gorman 2022[s21]		Uncertain evidence															
Gutiérrez Valencia 2016[s22]				Significant improvement			Uncertain evidence	Uncertain evidence		Uncertain evidence		No difference	Uncertain evidence	Improved	Effective		
Hasan Ibrahim	Uncertain evidence	Uncertain evidence			Uncertain evidence	Uncertain evidence		No significant difference					No significant difference	No significant difference			

2021[s23]																	
Hatah 2014[s24]						No significant difference	No significant difference [OR: 1.50, (0.65 to 3.46), I^2: 82%]	No significant difference					No significant difference [OR: 0.69 (0.39, 1.21), I^2: 73%]		No significant difference		
Hikaka 2019[s25]					Uncertain evidence	Improvement		Significant improvement						No significant difference		Patients were satisfied	
Hill-Taylor 2016[s26]		Significant reduction [OR: 2.98 (1.30 to 6.83), I^2: 86.7%]	Significant reduction	Improved						Uncertain evidence		Uncertain evidence	No significant difference		Improvement		
Huiskes 2017[s27]	Medication review: greater reduction or a smaller increase	Reduction		Uncertain evidence	Reduction	Uncertain evidence	No significant difference [RR: 0.94 (0.76 to 1.17), I^2: 22%]	No significant difference	No significant difference	Significant reduction [RR: 0.68 (0.52 to 0.90), I^2: 41%]	No significant difference		No significant difference [RR: 0.94 (0.82 to 1.08), I^2: 42.3%]		Uncertain evidence		
Ibrahim 2021[s28]	Reduction	Significant reduction			Significant reduction		No significant	No significant		Uncertain evidence	No significant		No significant		Cost-effective	Well accepted	Well accepted

							difference	difference			difference		difference				
Johansson 2016[s29]	Uncertain evidence						No significant difference [OR: 1.02 (0.84 to 1.23), I^2: 8%]						No significant difference [RR: 0.92 (0.50 to 1.70)]				
Kim 2024[s30]							Significant reduction [RR: 0.57 (0.37 to 0.88), I^2: 0%]	No significant difference [SMD: 0.36 (-0.01 to 0.73), I^2: 86.9%]					Significant reduction [RR: 0.57 (0.41 to 0.81), I^2: 72.4%]				
Kua 2019[s31]		Significant reduction [OR: 0.41 (0.19 to 0.89), I^2: 89%]					Reduction [OR: 0.90 (0.82 to 0.99), I^2: 51%]			No significant difference [OR: 0.85 (0.73 to 1.00), I^2: 14%]			No significant difference [OR: 0.72 (0.31 to 1.66), I^2: 68%]				
Laberge 2021[s32]	No significant difference	Reduction			Reduction								Uncertain evidence		Benefits might outweigh the implementation cost		

Lee 2021[s33]										No difference in fall rate [RR: 0.98 (0.63 to 1.51), I^2: 83%], fall incidence [RR: 1.04 (0.86 to 1.26)], or fall-related injuries [RR: 0.89 (0.57 to 1.39)]							
Liu 2024[s34]	No significant difference in hospital [MD: 0.02 (-0.28 to 0.32), I^2: 0] or community [MD: -0.03 (-0.08 to										Uncertain evidence			No significant improvement			

	0.01), I^2: 0)]																
Loganathan 2011[s35]	Uncertain evidence			Improved	No significant difference												
Maeda 2009[s36]	Significant reduction [median: 0.45 drugs (0.11 to 0.76)]																
Masnoon 2024[s37]	Uncertain evidence			Significant reduction	Significant reduction			Significant improvements	No significant difference	No significant difference	No significant difference		Significant reduction	Uncertain evidence	Cost-effective		
Mizokami 2019[s38]													Significant reduction in inpatients [RR: 0.89 (0.80 to 0.98), I^2: 9%] but not in outpatients [RR: 1.11 (0.99 to				

													1.24), I^2: 71%]				
Monteiro 2019[s39]	Uncertain evidence	Reduction		Improved drug appropriateness	Significant reduction	Adherence rate: 33 to 55%	No significant reduction					No significant reduction			95% probability of cost-effectiveness		Uncertain evidence
Mucherino 2022[s40]		Reduction												Uncertain evidence	Inconsistent (50% of the studies: not cost-effective; 25%: cost reduced)		
Nakham 2020[s41]	No significant reduction	Reduction													Uncertain evidence		
Njoku 2024[s42]	Significant reduction						Significant reduction	Reduction		Uncertain evidence							
Omuya 2023[s43]	Reduction						Uncertain evidence	Significant improvement		Uncertain evidence			Uncertain evidence		Improvement in healthcare cost		
O'Shea 2022[s44]													Reduction	Could reduce	Improvement in healthcare cost		

Page 2016[s45]	Significant reduction [MD: -0.99 (-1.83 to -0.14)]	Significant reduction [MD: -0.49 (-0.70 to -0.28)]			No significant difference		Non-randomized studies: significant reduction [OR: 0.32 (0.17 to 0.60)]; RCTs: no significant reduction [OR: 0.82 (0.61 to 1.11)]	No significant difference									
Patterson 2014[s46]		No significant difference [MD: -0.1 (-0.28 to 0.09), I^2: 89%)]		Significant reduction [MD: -6.78 (-12.34 to -1.22), I^2: 96%]	Uncertain evidence	Improvement		No difference					Uncertain evidence				
Pazan 2021[s47]									Uncertain evidence								
Pazan 2022[s48]											Uncertain evidence						

Quek 2024[s49]	Significant reduction [MD: -0.50 (-0.88 to -0.13), I^2: 94%]	Significant reduction [MD: 0.34 (0.06 to 0.63), I^2: 97%]		No significant difference [MD: -0.08 (-0.35 to 0.19), I^2: 73%]	No significant difference [OR: 1.11 (0.64 to 1.91), I^2: 79%]		No significant difference in RCTs [OR: 0.96 (0.84 to 1.09), I^2: 3%] or in non-randomized studies [OR: 0.70 (0.36 to 1.38), I^2: 41%]	No significant difference		No significant difference [OR: 0.88 (0.66 to 1.17), I^2: 49%; MD: -0.01 (-0.09 to 0.07), I^2: 0%]	No significant difference		No significant difference [OR: 0.99 (0.82 to 1.21), I^2: 70%]	Uncertain evidence			
Rao 2024[s50]		Reduction for short-term (≤ 4 months)															
Reeve 2022[s51]						Improvement	Uncertain evidence	Uncertain evidence	Improvements	Uncertain evidence			Uncertain evidence			Clinicians accepted	Patients accepted
Rodrigues 2022[s52]		Medication review: reduction															
Romano 2022[s53]								Small but significant gain		Small but significant reduction					Most interventions were cost-effective		

Roncal-Belzunce 2024[s54]	Significant reduction [MD: -0.57 (-0.81 to -0.32), I^2: 79%]	No significant difference [MD: -0.32 (-0.69 to 0.04), I^2: 90%]			Significant reduction [MD: -0.45 (-0.71 to -0.18), I^2: 37%]	Significant improvement [RR: 1.14 (1.04 to 1.24), I^2: 9%]	No significant difference [RR: 0.82 (0.57 to 1.17), I^2: 0%]	Non-significant difference [SMD: 0.18 (-0.08 to 0.44), I^2: 87%]	Uncertain evidence	No significant difference	Better outcome		No significant difference [RR: 0.82 (0.50 to 1.34), I^2: 18%]		Three of four studies show ed lower medication costs		No significant difference
Saeed 2022[s55]	Significant reduction	Significant reduction		Significant improvements	No significant difference		No significant difference	No significant difference		No significant difference					Deprescribing: cost saving		
Seppala 2022[s56]										No significant difference in community [RR: 1.05 (0.85 to 1.29), I^2: 0%] or hospital [RR: 0.97 (0.74 to 1.28), I^2: 15%]							
Shafiee Hanjani 2019[s57]	Uncertain evidence	Uncertain evidence		Uncertain evidence	Uncertain evidence		Uncertain evidence	Uncertain evidence		Uncertain evidence				Uncertain evidence			

Shrestha 2020[s58]	Uncertain evidence	Significant reduction	Significant reduction	Significant reduction	Reduction		Uncertain evidence	Uncertain evidence	No significant difference	Uncertain evidence	No significant difference				Significant improvement		
Shrestha 2021[s59]					No significant difference		Significant reduction (RR: 0.59 (0.44-0.79), I^2: 0%]	No significant difference	No significant difference	No significant difference [RR: 0.87 (0.65-1.17), I2: 0%]	No significant difference		No significant difference [RR: 1.12 (0.74-1.69), I^2: 6%]				
Sirois 2024[s60]	Uncertain evidence	Uncertain evidence					Uncertain evidence										
Tasai 2021[s61]						Significant improvement		Uncertain evidence					Trend toward reduction				
Tecklenborg 2020[s62]							No significant difference [RR: 1.44 (0.96 to 2.15), I^2: 0%]	No significant difference [SMD: -0.11 (-0.24 to 0.01), I^2: 0%)	No significant difference [SMD: 0.02 (-0.18 to 0.23), I^2:27.2%]				No significant reduction [RR: 0.87 (0.52 to 1.47), I^2:0%]				
Thillainadesan 2018[s63]		Uncertain evidence		Uncertain evidence	Uncertain evidence		No significant difference	Uncertain evidence	significant improvement	Significant reduction			No significant difference				

Thomas 2014[s64]													No significant reduction by hospital pharmacists [RR: 0.97 (0.88 to 1.07)] or community pharmacists [RR: 1.07 (0.96 to 1.20), I^2: 33%]				
Tjia 2013[s65]	Significant reduction	Significant reduction		Significant reduction	Significant reduction											63-92%	
Ulley 2019[s66]	Overall reduction					Uncertain evidence	No difference	Uncertain evidence					Uncertain evidence				
Vaismoradi 2024[s67]	Reduction					Improvement		Improved	Improved						Improved		Improved
Walsh 2016[s68]				Significant reduction [MD: -	Detected significantly more		No significant						No significant				

				5.27 (-8.44 to -2.11), I^2: 93%]	frequently		difference						difference				
Williams 2008[s69]						Weak evidence	No difference	Uncertain evidence						No difference	Could reduce cost		
Zhou 2023[s70]		Significant reduction [RR: 0.69 (0.53 to 0.90), I^2: 93%]	Significant reduction [RR: 0.28 (0.12 to 0.64), I^2: 91%]		Significant reduction [RR: 0.61 (0.48 to 0.78), I^2: 0%)	Significant improvement [RR: 1.26 (1.02 to 1.55), I^2: 9%]	No significant difference [RR: 0.97 (0.89 to 1.07), I^2: 38%]	No significant difference		No significant difference [RR: 0.94 (0.84 to 1.06), I^2: 0%]			No significant difference [RR: 0.95 (0.90 to 1.00), I^2: 9%]	No difference			
Zhou 2023[s71]	Significant reduction [MD: -0.94 (-1.51 to -0.36), I^2: 0%]	Significant reduction in incidence [OR: 0.51 (0.42 to 0.62), I^2: 0%] or number: [MD: -0.41 (-0.51 to -0.31), I^2: 0%]					No significant reduction [OR: 0.93 (0.64 to 1.37), I^2: 0%]			No significant reduction			Significant reduction [OR: 0.58 (0.36 to 0.92), I^2: 0%]				

ARD: absolute risk difference, ARR: absolute risk reduction, HR: hazard ratio, MD: mean difference, SMD: standardized mean difference, OR: odds ratio, RCT: randomized controlled trials, RR: relative risk or risk ratio, WMD: weighted mean difference.
The numbers inside the round brackets indicate the lower and upper limits of 95% confidence intervals. The cells shaded with light brown, grey, and light green colors signify negative, inconsistent/uncertain, and positive results, respectively. The font colors red, orange, green, and black signify low/very low, moderate, high GRADE (Grading of Recommendations, Assessment, Development, and Evaluations) of evidence, and no reported GRADE of evidence respectively as mentioned in the original studies.

Table s5. Assessment of the credibility of evidence for each outcome.

**Outcome**	**Level of Evidence**
Reduction in the number of medications	Convincing evidence
Reduction in potentially inappropriate medications (PIMs)	Convincing evidence
Improvement in medication appropriateness	Convincing evidence
Enhancement in medication adherence	Convincing evidence
Reduction in falls	Convincing evidence
Reduction in adverse drug reactions or drug-related problems	Highly suggestive evidence
Reduction in hospital visits, admissions, and re-admissions	Highly suggestive evidence
Improvement in quality of life	Suggestive evidence
Improvement in acceptability of the intervention among physicians	Suggestive evidence
Improvement in acceptability of the intervention among patients	Suggestive evidence
Physical functions	Not significant
All-cause mortality	Weak evidence
Cognitive function	Weak evidence
Cost-effectiveness	Weak evidence
Duration of hospitalization	Weak evidence
Reduction in potential prescribing omissions (PPOs)	Weak evidence
Healthcare utilization	Weak evidence

Supplementary References

s1. Ali MU, Sherifali D, Fitzpatrick-Lewis D, et al. Polypharmacy and mobility outcomes. Mech Ageing Dev. 2020;192:111356.
s2. Alldred DP, Kennedy MC, Hughes C, et al. Interventions to optimise prescribing for older people in care homes. Cochrane Database Syst Rev. 2016;2:CD009095.
s3. Almutairi H, Stafford A, Etherton-Beer C, et al. Optimisation of medications used in residential aged care facilities: a systematic review and meta-analysis of randomised controlled trials. BMC Geriatr. 2020;20:236.
s4. Bloomfield HE, Greer N, Linsky AM, et al. Deprescribing for Community-Dwelling Older Adults: a Systematic Review and Meta-analysis. J Gen Intern Med. 2020;35:3323-3332.
s5. Bülow C, Clausen SS, Lundh A, et al. Medication review in hospitalised patients to reduce morbidity and mortality. Cochrane Database Syst Rev. 2023;1:CD008986.
s6. Burgos-Alonso N, Torrecilla M, Mendiguren A, et al. Strategies to Improve Therapeutic Adherence in Polymedicated Patients over 65 Years: A Systematic Review and Meta-Analysis. Pharmacy (Basel). 2024;12:35.
s7. Carollo M, Crisafulli S, Vitturi G, et al. Clinical impact of medication review and deprescribing in older inpatients: A systematic review and meta-analysis. J Am Geriatr Soc. 2024;72:3219-3238.
s8. Clarkson L, Hart L, Lam AK, et al. Reducing inappropriate polypharmacy for older patients at specialist outpatient clinics: a systematic review. Curr Med Res Opin. 2023;39:545-554.
s9. Clyne B, Fitzgerald C, Quinlan A, et al. Interventions to Address Potentially Inappropriate Prescribing in Community-Dwelling Older Adults: A Systematic Review of Randomized Controlled Trials. J Am Geriatr Soc. 2016;64:1210-1222.
s10. Cole JA, Gonçalves-Bradley DC, Alqahtani M, et al. Interventions to improve the appropriate use of polypharmacy for older people. Cochrane Database Syst Rev. 2023;10:CD008165.
s11. Cooper JA, Cadogan CA, Patterson SM, et al. Interventions to improve the appropriate use of polypharmacy in older people: a Cochrane systematic review. BMJ Open. 2015;5:e009235.
s12. Croke A, Cardwell K, Clyne B, et al. The effectiveness and cost of integrating pharmacists within general practice to optimize prescribing and health outcomes in primary care patients with polypharmacy: a systematic review. BMC Prim Care. 2023;24:41.
s13. Cross AJ, Elliott RA, Petrie K, et al. Interventions for improving medication-taking ability and adherence in older adults prescribed multiple medications. Cochrane Database Syst Rev. 2020;5:CD012419.
s14. Dalton K, O'Brien G, O'Mahony D, et al. Computerised interventions designed to reduce potentially inappropriate prescribing in hospitalised older adults: a systematic review and meta-analysis. Age Ageing. 2018;47:670-678.
s15. de Andrade FK, Ignacio Nunes RP, Barboza Zanetti MO, et al. Validated medication deprescribing instruments for patients with palliative care needs palliative care: A systematic review. Farm Hosp. 2024;48:T83-T89.
s16. Dongo A, Kantaris M. Assessing the effectiveness of pharmacistinitiated strategies on prescription errors and drug-associated problems among geriatric patients within a hospital setting: a systematic review. S Afr Pharm J. 2023;90:15a-15h.
s17. Earl TR, Katapodis ND, Schneiderman SR, et al. Using Deprescribing Practices and the Screening Tool of Older Persons' Potentially Inappropriate Prescriptions Criteria to Reduce Harm and Preventable Adverse Drug Events in Older Adults. J Patient Saf. 2020;16:S23-S35.
s18. Faulkner L, Hughes CM, Barry HE. Interventions to improve medicines optimisation in older people with frailty in primary care: a systematic review. Int J Pharm Pract. 2022;30:297-304.
s19. Forsetlund L, Eike MC, Gjerberg E, et al. Effect of interventions to reduce potentially inappropriate use of drugs in nursing homes: a systematic review of randomised controlled trials. BMC Geriatr. 2011;11:16.
s20. George J, Elliott RA, Stewart DC. A systematic review of interventions to improve medication taking in elderly patients prescribed multiple medications. Drugs Aging. 2008;25:307-324.
s21. Gorman A, Rankin A, Hughes C, et al. Theoretically derived interventions aimed at improving appropriate polypharmacy in primary care: A systematic review. Explor Res Clin Soc Pharm. 2022;7:100166.
s22. Gutiérrez Valencia M, Martínez Velilla N, et al. Intervenciones para optimizar el tratamiento farmacológico en ancianos hospitalizados: una revisión sistemática [Interventions to optimize pharmacologic treatment in hospitalized older adults: a systematic review]. Rev Clin Esp (Barc). 2016;216:205-221.
s23. Hasan Ibrahim AS, Barry HE, Hughes CM. A systematic review of general practice-based pharmacists' services to optimize medicines management in older people with multimorbidity and polypharmacy. Fam Pract. 2021;38:509-523.
s24. Hatah E, Braund R, Tordoff J, et al. A systematic review and meta-analysis of pharmacist-led fee-for-services medication review. Br J Clin Pharmacol. 2014;77:102-115.
s25. Hikaka J, Hughes C, Jones R, et al. A systematic review of pharmacist-led medicines review services in New Zealand - is there equity for Māori older adults? Res Social Adm Pharm. 2019;15:1383-1394.

s26. Hill-Taylor B, Walsh KA, Stewart S, et al. Effectiveness of the STOPP/START (Screening Tool of Older Persons' potentially inappropriate Prescriptions/Screening Tool to Alert doctors to the Right Treatment) criteria: systematic review and meta-analysis of randomized controlled studies. J Clin Pharm Ther. 2016;41:158-169.
s27. Huiskes VJ, Burger DM, van den Ende CH, et al. Effectiveness of medication review: a systematic review and meta-analysis of randomized controlled trials. BMC Fam Pract. 2017;18:5.
s28. Ibrahim K, Cox NJ, Stevenson JM, et al. A systematic review of the evidence for deprescribing interventions among older people living with frailty. BMC Geriatr. 2021;21:258.
s29. Johansson T, Abuzahra ME, Keller S, et al. Impact of strategies to reduce polypharmacy on clinically relevant endpoints: a systematic review and meta-analysis. Br J Clin Pharmacol. 2016;82:532-548.
s30. Kim IJ, Ryu G, Rhie SJ, et al. Pharmacist interventions in Asian healthcare environments for older people: a systematic review and meta-analysis on hospitalization, mortality, and quality of life. BMC Geriatr. 2024;24:513.
s31. Kua CH, Mak VSL, Huey Lee SW. Health Outcomes of Deprescribing Interventions Among Older Residents in Nursing Homes: A Systematic Review and Meta-analysis. J Am Med Dir Assoc. 2019;20:362-372.e11.
s32. Laberge M, Sirois C, Lunghi C, et al. Economic Evaluations of Interventions to Optimize Medication Use in Older Adults with Polypharmacy and Multimorbidity: A Systematic Review. Clin Interv Aging. 2021;16:767-779.
s33. Lee J, Negm A, Peters R, et al. Deprescribing fall-risk increasing drugs (FRIDs) for the prevention of falls and fall-related complications: a systematic review and meta-analysis. BMJ Open. 2021;11:e035978.
s34. Liu BM, Redston MR, Fujita K, et al. The Impact of Deprescribing Interventions on the Drug Burden Index and Other Outcomes: A Systematic Review. J Am Med Dir Assoc. 2024;25:105021.
s35. Loganathan M, Singh S, Franklin BD, et al. Interventions to optimise prescribing in care homes: systematic review. Age Ageing. 2011;40:150-162.
s36. Maeda K. Systematic review of the effects of improvement of prescription to reduce the number of medications in the elderly with polypharmacy. Yakugaku Zasshi. 2009;129:631-645.
s37. Masnoon N, George C, Lo S, et al. The outcomes of considering goals of care in medication reviews for older adults: a systematic review. Expert Rev Clin Pharmacol. 2024;17:33-56.
s38. Mizokami F, Mizuno T, Kanamori K, et al. Clinical medication review type III of polypharmacy reduced unplanned hospitalizations in older adults: A meta-analysis of randomized clinical trials. Geriatr Gerontol Int. 2019;19:1275-1281.
s39. Monteiro L, Maricoto T, Solha I, et al. Reducing Potentially Inappropriate Prescriptions for Older Patients Using Computerized Decision Support Tools: Systematic Review. J Med Internet Res. 2019;21:e15385.
s40. Mucherino S, Casula M, Galimberti F, et al.; On Behalf Of The Edu Re Drug Group. The Effectiveness of Interventions to Evaluate and Reduce Healthcare Costs of Potentially Inappropriate Prescriptions among the Older Adults: A Systematic Review. Int J Environ Res Public Health. 2022;19:6724.
s41. Nakham A, Myint PK, Bond CM, et al. Interventions to Reduce Anticholinergic Burden in Adults Aged 65 and Older: A Systematic Review. J Am Med Dir Assoc. 2020;21:172-180.e5.
s42. Njoku CM, Barnett F, Fiatarone et al. Multifaceted Interventions Inclusive of Medication Optimization for Frailty in Aged Care: A Systematic Review. J Am Med Dir Assoc. 2024;25:105111.
s43. Omuya H, Nickel C, Wilson P, et al. A systematic review of randomised-controlled trials on deprescribing outcomes in older adults with polypharmacy. Int J Pharm Pract. 2023;31:349-368.
s44. O'Shea J, Ledwidge M, Gallagher J, et al. Pharmacogenetic interventions to improve outcomes in patients with multimorbidity or prescribed polypharmacy: a systematic review. Pharmacogenomics J. 2022;22:89-99. Erratum in: Pharmacogenomics J. 2022;22:146.
s45. Page AT, Clifford RM, Potter K, et al. The feasibility and effect of deprescribing in older adults on mortality and health: a systematic review and meta-analysis. Br J Clin Pharmacol. 2016;82:583-623.
s46. Patterson SM, Cadogan CA, Kerse N, et al. Interventions to improve the appropriate use of polypharmacy for older people. Cochrane Database Syst Rev. 2014;10:CD008165.
s47. Pazan F, Petrovic M, Cherubini A, et al. Current evidence on the impact of medication optimization or pharmacological interventions on frailty or aspects of frailty: a systematic review of randomized controlled trials. Eur J Clin Pharmacol. 2021;77:1-12.
s48. Pazan F, Petrovic M, Cherubini A, et al. A Systematic Review of the Current Evidence from Randomised Controlled Trials on the Impact of Medication Optimisation or Pharmacological Interventions on Quantitative Measures of Cognitive Function in Geriatric Patients. Drugs Aging. 2022;39:863-874.
s49. Quek HW, Page A, Lee K, et al. The effect of deprescribing interventions on mortality and health outcomes in older people: An updated systematic review and meta-analysis. Br J Clin Pharmacol. 2024;90:2409-2482.
s50. Rao P, Hung A. Impact of medication therapy management programs on potentially inappropriate medication use in older adults: A systematic review. J Manag Care Spec Pharm. 2024;30:3-14.
s51. Reeve J, Maden M, Hill R, et al. Deprescribing medicines in older people living with multimorbidity and polypharmacy: the TAILOR evidence synthesis. Health Technol Assess. 2022;26:1-148.
s52. Rodrigues DA, Plácido AI, Mateos-Campos R, et al. Effectiveness of Interventions to Reduce Potentially Inappropriate Medication in Older Patients: A Systematic Review. Front Pharmacol. 2022;12:777655.

s53. Romano S, Figueira D, Teixeira I, et al. Deprescribing Interventions among Community-Dwelling Older Adults: A Systematic Review of Economic Evaluations. Pharmacoeconomics. 2022;40:269-295.
s54. Roncal-Belzunce V, Gutiérrez-Valencia M, Leache L, et al. Systematic review and meta-analysis on the effectiveness of multidisciplinary interventions to address polypharmacy in community-dwelling older adults. Ageing Res Rev. 2024;98:102317.
s55. Saeed D, Carter G, Parsons C. Interventions to improve medicines optimisation in frail older patients in secondary and acute care settings: a systematic review of randomised controlled trials and non-randomised studies. Int J Clin Pharm. 2022;44:15-26.
s56. Seppala LJ, Kamkar N, van Poelgeest EP, et al.; Task Force on Global Guidelines for Falls in Older Adults. Medication reviews and deprescribing as a single intervention in falls prevention: a systematic review and meta-analysis. Age Ageing. 2022 Sep 2;51:afac191. Erratum in: Age Ageing. 2023;52:afad188.
s57. Shafiee Hanjani L, Long D, Peel NM, et al. Interventions to Optimise Prescribing in Older People with Dementia: A Systematic Review. Drugs Aging. 2019;36:247-267.
s58. Shrestha S, Poudel A, Steadman K, et al. Outcomes of deprescribing interventions in older patients with life-limiting illness and limited life expectancy: A systematic review. Br J Clin Pharmacol. 2020;86:1931-1945.
s59. Shrestha S, Poudel A, Cardona M, et al. Impact of deprescribing dual-purpose medications on patient-related outcomes for older adults near end-of-life: a systematic review and meta-analysis. Ther Adv Drug Saf. 2021;12:20420986211052343.
s60. Sirois C, Gosselin M, Laforce C, et al. How does deprescribing (not) reduce mortality? A review of a meta-analysis in community-dwelling older adults casts uncertainty over claimed benefits. Basic Clin Pharmacol Toxicol. 2024;134:51-62.
s61. Tasai S, Kumpat N, Dilokthornsakul P, et al. Impact of Medication Reviews Delivered by Community Pharmacist to Elderly Patients on Polypharmacy: A Meta-analysis of Randomized Controlled Trials. J Patient Saf. 2021;17:290-298.
s62. Tecklenborg S, Byrne C, Cahir C, et al. Interventions to Reduce Adverse Drug Event-Related Outcomes in Older Adults: A Systematic Review and Meta-analysis. Drugs Aging. 2020;37:91-98. Erratum in: Drugs Aging. 2020;37:147.
s63. Thillainadesan J, Gnjidic D, Green S, et al. Impact of Deprescribing Interventions in Older Hospitalised Patients on Prescribing and Clinical Outcomes: A Systematic Review of Randomised Trials. Drugs Aging. 2018;35:303-319.
s64. Thomas R, Huntley AL, Mann M, et al. Pharmacist-led interventions to reduce unplanned admissions for older people: a systematic review and meta-analysis of randomised controlled trials. Age Ageing. 2014;43:174-187.
s65. Tjia J, Velten SJ, Parsons C, et al. Studies to reduce unnecessary medication use in frail older adults: a systematic review. Drugs Aging. 2013;30:285-307.
s66. Ulley J, Harrop D, Ali A, et al. Deprescribing interventions and their impact on medication adherence in community-dwelling older adults with polypharmacy: a systematic review. BMC Geriatr. 2019;19:15.
s67. Vaismoradi M, Mardani A, Crespo ML, et al. An integrative systematic review of nurses' involvement in medication deprescription in long-term healthcare settings for older people. Ther Adv Drug Saf. 2024;15:20420986241289205.
s68. Walsh KA, O'Riordan D, Kearney PM, et al. Improving the appropriateness of prescribing in older patients: a systematic review and meta-analysis of pharmacists' interventions in secondary care. Age Ageing. 2016;45:201-209.
s69. Williams A, Manias E, Walker R. Interventions to improve medication adherence in people with multiple chronic conditions: a systematic review. J Adv Nurs. 2008;63:132-143.
s70. Zhou D, Chen Z, Tian F. Deprescribing Interventions for Older Patients: A Systematic Review and Meta-Analysis. J Am Med Dir Assoc. 2023;24:1718-1725.
s71. Zhou S, Li R, Zhang X, et al. The effects of pharmaceutical interventions on potentially inappropriate medications in older patients: a systematic review and meta-analysis. Front Public Health. 2023;11:1154048.
